# Inferences on specificity recognition at the *Malus×domestica* gametophytic self-incompatibility system

**DOI:** 10.1038/s41598-018-19820-1

**Published:** 2018-01-29

**Authors:** Maria I. Pratas, Bruno Aguiar, Jorge Vieira, Vanessa Nunes, Vanessa Teixeira, Nuno A. Fonseca, Amy Iezzoni, Steve van Nocker, Cristina P. Vieira

**Affiliations:** 1 Instituto de Biologia Molecular e Celular (IBMC), Universidade do Porto, Rua Alfredo Allen 208, 4200-135 Porto, Portugal; 2 Instituto de Investigação e Inovação em Saúde, Universidade do Porto, Rua Alfredo Allen 208, 4200-135 Porto, Portugal; 3 European Bioinformatics Institute (EMBL-EBI,) Welcome Trust Genome Campus, CB10 1SD Cambridge, United Kingdom; 4 Michigan State University, East Lansing, MI 48824-1325 USA

## Abstract

In *Malus* × *domestica* (Rosaceae) the product of each *SFBB* gene (the pollen component of the gametophytic self-incompatibility (GSI) system) of a *S*-haplotype (the combination of pistil and pollen genes that are linked) interacts with a sub-set of non-self S-RNases (the pistil component), but not with the self S-RNase. To understand how the *Malus* GSI system works, we identified 24 *SFBB* genes expressed in anthers, and determined their gene sequence in nine *M. domestica* cultivars. Expression of these *SFBBs* was not detected in the petal, sepal, filament, receptacle, style, stigma, ovary or young leaf. For all *SFBBs* (except *SFBB15)*, identical sequences were obtained only in cultivars having the same *S-RNase*. Linkage with a particular *S-RNase* was further established using the progeny of three crosses. Such data is needed to understand how other genes not involved in GSI are affected by the *S*-locus region. To classify SFBBs specificity, the amino acids under positive selection obtained when performing intra-haplotypic analyses were used. Using this information and the previously identified S-RNase positively selected amino acid sites, inferences are made on the S-RNase amino acid properties (hydrophobicity, aromatic, aliphatic, polarity, and size), at these positions, that are critical features for GSI specificity determination.

## Introduction

Gametophytic self-incompatibility (GSI), the most common reproductive system in flowering plants (see Fig. 1 in Igic *et al*.^1^), is a pre-zygotic genetic mechanism that prevents self-fertilization and promotes out-crossing, by enabling the pistil to reject pollen from genetically related individuals^2^. In this system, to preserve functional incompatibility, there are two distinct components, one that determines the pistil specificity and another that determines the pollen specificity, called *S-* genes. The locus that contains the genes determining GSI specificity is called the *S*-locus.

The pistil specificity component in Rosaceae, Rubiaceae, Solanaceae and Plantaginaceae species, is an extracellular ribonuclease, called S-RNase^3–5^. Since RNase activity is needed for inhibition of self-pollen tube growth^6^, it has been assumed that degradation of pollen tube RNAs in the self-pollen tube is part of the biochemical mechanism of self-incompatibility (SI). According to the phylogeny of this gene and the conserved structure (conserved and hypervariable regions, intron number and position) *RNase* based GSI has evolved only once, before the separation of the Asterideae and Rosideae, about 120 million years ago^5,7–9^. Nevertheless, in Rosaceae, Pyrinae (*Malus*, *Pyru*s and *Sorbus*) and *Prunus S-RNase* based GSI evolved from paralogous genes, according to phylogenetic analyses of the *S-RNase* and *S*-pollen lineage genes. *Malus* and *Prunus* GSI genes belong to distinct gene lineages, and only *Prunus* GSI -lineage genes are present in *Fragaria*, that is an out-group to both species^10^.

The pollen specificity component encodes a F-box protein(s), and varies from one gene in *Prunus* (called *SFB*, S-haplotype specific F-box gene)^11–18^, to multiple genes in *Malus*, *Pyrus*, *Sorbus* (called *SFBB*s, *S*-locus F-box brothers), *Petunia*, and *Nicotiana* (Solanaceae; called *SLF*s, S-locus F-box)^19–28^. *Prunus SFB* and *SFBBs*/*SLFs* genes are not orthologous^10,25,28–31^. Therefore, it is not surprising that in *Prunus* a self-recognition mechanism is used for S-RNase inhibition^13,32,33^, while in the species presenting multiple *S*-pollen genes, each *S*-protein recognizes and interacts with a sub-set of non-self S-RNases, to mediate their degradation^19–22,24,34–36^. In *Petunia*, transgenic experiments were performed to address the function of genes involved in pollen specificity^37^. Diploid pollen carrying two different functional *S*-haplotypes or haploid pollen that carries a duplicated *S*-locus region of a different *S*-haplotype caused breakdown of self-incompatibility^38–40^. Nevertheless, this was not always observed, implying additional *S*-pollen genes determining pollen specificity^24^. Furthermore, coimmunoprecipitation results showed non-self interactions between *S*-pollen proteins and the S-RNases in SI responses^24^. These observations led to the collaborative non-self recognition model, that takes into account the involvement of multiple *S*-pollen proteins in pollen specificity. In Pyrinae (*Malus*, *Pyrus*, and *Sorbus*), such transformation methods are not possible since these species are trees. Nevertheless, in *Malus*, yeast two-hybrid analysis indicated that SFBBs interact mostly with non-self S-RNases^41^. Other sequences assigned as SFBB-like, however, also show a similar pattern. These SFBB-like sequences may be encoded by *SFBB* genes since they are expressed in pollen only and are located in the vicinity of the *S-RNase*, some of them in between recognized *SFBB* genes. They have been assigned as SFBB-like because the authors were not able to show *S-*haplotype linkage. This, however, may be due to difficulties in designing specific primers, since *SFBB* genes can present low nucleotide divergence. Moreover, using the predicted tertiary structure of S-RNases and SFBBs and their binding energies, based on the Wilcoxon rank-sum test, when the hypervariable region of the S-RNase is considered, it has been shown that SFBBs of a *S-*haplotype interact more strongly with non-self than with self S-RNases^42^. Therefore, it seems that in *Malus* the GSI system works in a similar way to that of *Petunia*.

Because the selective pressures in recognition mechanisms with one or multiple *S-*pollen genes are different, the *S*-pollen genes show distinct evolutionary patterns. In *Prunus* the two *S*-genes must co-evolve for specificity recognition and, thus, both genes present similar levels of diversity and number of amino acids under positive selection (those that in principle are involved in specificity determination)^12,43^. In the collaborative non-self -recognition model each *S*-pollen protein recognizes a sub-set of non-self- S-RNases, and levels of diversity at these genes are, at least 2.5 times lower than those of the *S*-pistil gene^20,22,24,28,34,44^. Levels of intra-haplotype divergence are, however, similar to the *S-RNase* diversity^22,34^, and amino acids under positive selection have been identified in *Sorbus* (Pyrinae, Rosaceae) when intra haplotypic analyses are performed^22^.

Essential for the understanding of the collaborative non-self -recognition model is knowledge of how many *S*-pollen genes exist in a *S*-haplotype. In *Petunia*, anthers transcriptomes of two homozygous plants (*S2S2*, and *S3S3*) revealed 17 *S*-pollen genes for both *S*-haplotypes^21^. 10 of these *S*-pollen genes were previously identified^24,30,45–47^, and for eight, transgenic functional assays, have been performed to show that they are involved in *S*-pollen specificity^21,24,37^. Moreover, all 17 SLF proteins of both *S*-haplotypes, using co-immunoprecipitation and mass spectrometry assays, have been shown to be assembled into similar canonical SCF complexes to the eight SLFs confirmed to be involved in GSI^36^. Furtermore, in *Petunia*, the study of 12 homozygous plants, using a combination of next-generation sequencing (from mature pollen and unopened mature anthers) and PCR techniques, revealed that the number of *SLF* genes per *S*-haplotype varies from 16 to 20^20^. These genes define 18 specificity types, and within each type, variation in terms of copy number and amino acid sequence polymorphism was found. Then, variation was used to predict the target S-RNase(s) of each type of SLF, using the rule put forth by Kubo *et al*.^20^: “Sx-RNase is a target of SLFn if the Sx-allele of SLFn is diverged or deleted”. For eight *S*-haplotypes, predictions were made regarding the SLF types that recognise seven of the S-RNases. Five of these predictions are supported by experimental data.

In Pyrinae (*Malus*, *Pyrus*, and *Sorbus*), 16 *SFBB*-like genes have been characterized from the sequencing of both BAC clones containing the *S*-locus, and PCR products obtained from genomic DNA using primers for conserved regions^22,23,27,44,48,49^. All these genes, as expected for *S*-pollen genes, are expressed in pollen only, and for all, except *SFBB15*, linkage with the *S-RNase* has been confirmed. Because of the methodologies used, the number of *SFBB*s in Pyrinae could be underestimated. Such data is needed to determine the size of the *S*-locus region and its effect on other genes unrelated to self-incompatibility that are located in the same region^50^. Therefore, in this work we sought to determine the number of *SFBB*s associated with *S*-haplotypes in *M. domestica* and to use this information to provide insights into GSI in *M. domestica* by addressing how copy number variation and amino acid sequence polymorphism at the amino acids under positive selection can be used to predict *S*-pollen specificity. Furthermore, we address which S-RNase amino acid characteristics, at those sites under positive selection, are involved in *S*-pollen specificity recognition. To identify the *SFBB*s, we used an approach similar to that used in *Petunia*, that was a combination of anthers transcriptome of nine *M. domestica* cultivars [‘Fuji’ (*S1, S9*), ‘Northern Spy’ (*S1*, *S3*), ‘Gala’ (*S2, S5*), ‘Golden Delicious’ (*S2, S3*), ‘Honeycrisp’ (*S2, S24*), ‘Idared’ (*S3, S7*), ‘Red Delicious’ (*S9, S28*), ‘McIntosh’ (*S10, S25*), and ‘Empire’ (*S10, S28*)] covering 10 *S*-haplotypes, and a PCR approach using genomic DNA to determine the number of *SFBB* genes in *Malus*.

## Results

### Assessing Transcriptome Coverage

Because the main goal of this work was to identify as many as possible candidate *SFBB* genes involved in pollen specificity through transcriptome sequencing of anthers, we first assessed the coverage of the transcriptomes used (Supplementary Table S1). According to the accumulation curve obtained for the nine anthers transcriptomes, the number of expressed *Malus* CDS detected in the sample increases at a slower rate after 6000000 paired reads (Supplementary Fig. S1), suggesting that the sampling is sufficient for the discovery of new *SFBB* genes. Moreover, the annotated *SFBB* genes on the *M. domestica* genome^50^ are identified in the anthers transcriptomes having *S2-* or *S3*-haplotypes (Supplementary Table S2), providing additional support that the coverage of the transcriptomes is sufficient for the identification of new *SFBB* genes. Furthermore, the 13 *S3-*, 14 *S9-*, and the six *S10-*haplotype *SFBB* genes previously reported^23,27,44^, are identified in anthers transcriptomes of the cultivars having these *S*-haplotypes (Supplementary Fig. S2), suggesting that the transcriptome coverage is enough for the identification of new *SFBB* genes.

**Figure 1:**
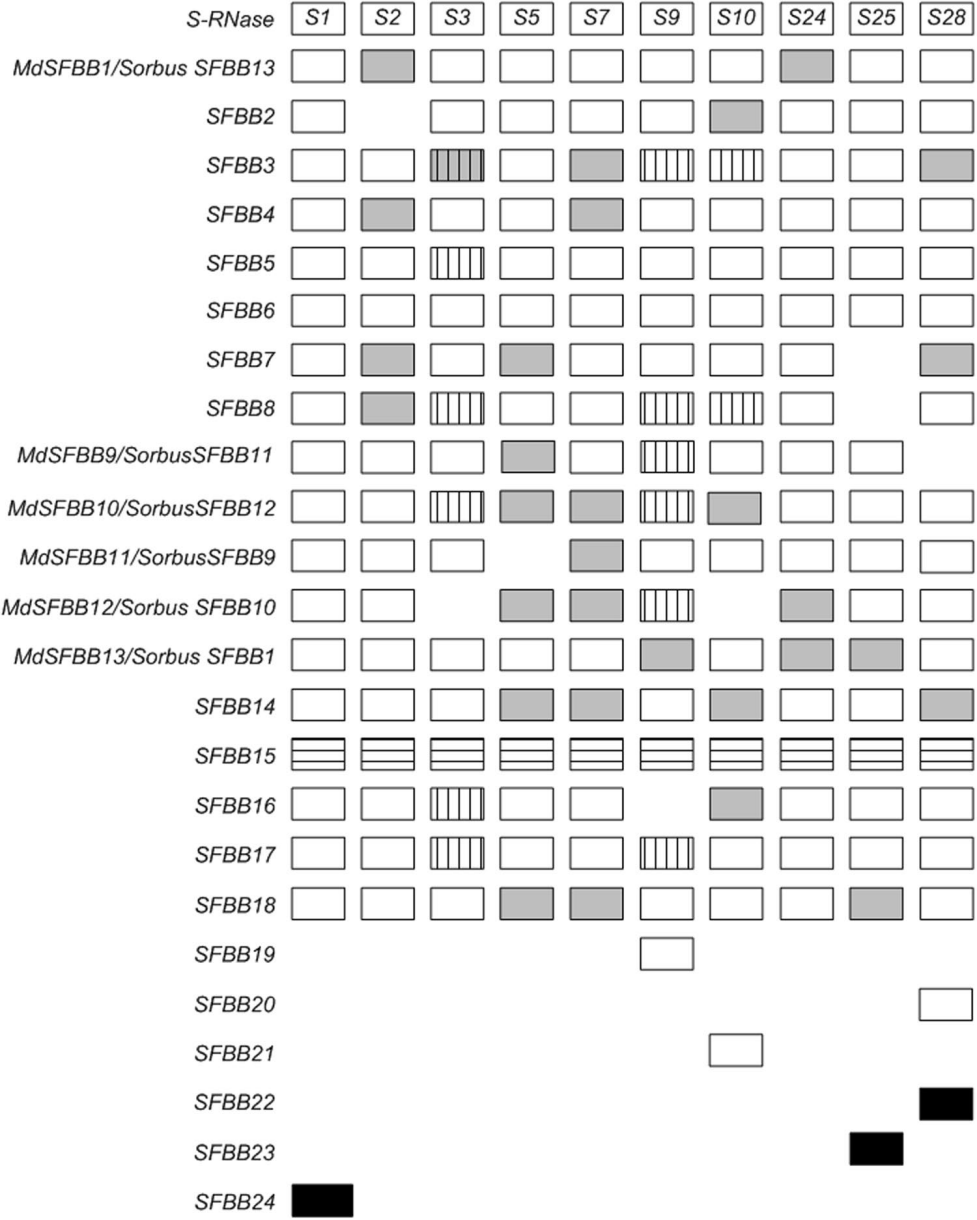
*SFBB* genes in the 10 *S*-haplotypes analysed. White boxes represent sequences obtained using primers SFBBgenF and SFBBgenR, grey boxes represent sequences obtained with specific primers for that particular gene, black boxes represent sequences obtained from Edena contigs. Boxes with vertical lines represent sequences described in the literature^23,27,44^ not amplified with primers SFBBgenF and SFBBgenR. Boxes with horizontal lines represent sequences that are identical in cultivars not sharing a *S-RNase*. The star indicates a *SFBB* sequence that presents stop codons in the putative coding region, obtained from ‘Golden Delicious’ (*S2, S3*), and ‘Honeycrisp’ (*S2, S24*), that is also present in the *Malus* genome (NW_007545880.1- 1139053… 1137851).

### Identifying *SFBB* genes from Edena assemblies

When the 33 *SFBB* sequences from *S3-, S9-*, and *S10-*haplotype^23,27,44^ were searched in the Trinity (Supplementary Table S3) and Edena (Supplementary Table S4) transcriptome assemblies of ‘Golden Delicious’ (*S2, S3*), ‘Northern Spy’ (*S1*, *S3*), ‘Idared’ (*S3, S7*), ‘Fuji’ (*S1, S9*) and ‘Red Delicious’ (*S9, S28*), ‘McIntosh’ (*S10, S25*) and ‘Empire’ (*S10, S28*), as described in Material and Methods, we found contigs for 28 and 26 *SFBB* sequences, respectively (Table 1). These sequences cover all *SFBB* genes described in the literature^23,27,44^. Nevertheless, these were smaller than 190 bp in the Trinity assembly, and with an average size of 697 bp for the Edena transcriptome assembly. It should be noted that the transcriptomes obtained are from heterozygous individuals (Material and Methods). Given the high level of sequence similarity between *SFBB* genes (Aguiar *et al*.^22^, and references therein), it is possible, that ambiguities arise during assembly giving rise to short contigs. Nevertheless, although only 78% of the *SFBB* alleles reported in the literature are represented in the two transcriptome assemblies, we could find at least one allele for all *SFBB* genes (Table 1).

**Table 1:** Size, in bp, of longest sequence in the Trinity (in bold) and Edena datasets derived from seven *M. domestica* cultivars that match the 33 *SFBB* sequences reported for the *S3-*, *S9-*, and *S10-*haplotypes^23,27,44^.

Gene	*S3*-haplotype			*S9*-haplotype		*S10*-haplotype	
	GD	NS	Idared	RD	Fuji	Mc	Empire
*MdSFBB1*/*SorbusSFBB13*	**144** (2)	—	**254** (1)	—	—	—	—
	556 (2)	—	601 (3)	905 (2)	408 (1)	—	—
*SFBB2*	**156** (1)	**122** (2)	—	**442** (2)	**285** (4)	n.a.	n.a.
	746 (1)	463 (1)	—	1191 (3)	—	n.a.	n.a.
*SFBB3*	**159** (1)	**108** (2)	**183** (2)	**255** (2)	**186** (1)	—	—
	—	—	—	542 (1)	—	—	—
*SFBB4*	—	—	**114** (2)	**183** (1)	—	**259** (1)	—
	781 (2)	546 (2)	1141 (4)	693 (2)	859 (3)	537 (2)	1049 (5)
*SFBB5*	**102** (1)	**117** (1)	**169** (2)	—	**151** (1)	n.a.	n.a.
	—	238 (1)	—	654 (2)	1175 (3)	n.a	n.a
*SFBB6*	—	**151** (1)	—	**278** (2)	**157** (1)	**179** (2)	—
	—	—	—	467 (1)	352 (1)	904 (5)	540 (2)
*SFBB7*	—	—	—	**106** (1)	—	n.a.	n.a.
	324 (2)	1069 (3)	647 (3)	860 (2)	647 (2)	n.a.	n.a.
*SFBB8*	**136** (2)	**141** (5)	**108** (1)	**323** (3)	**252** (1)	**436** (4)	**181** (4)
	—	—	—	138 (1)	—	—	—
*MdSFBB9*/*SorbusSFBB11*	**151** (1)	—	**150** (1)	**163** (1)	—	**105** (1)	**105** (1)
	840 (3)	—	1031 (4)	1171 (4)	710 (3)	747 (3)	1170 (4)
*MdSFBB10*/*SorbusSFBB12*	**153** (2)	**258** (1)	**156** (1)	**157** (2)	—	**101** (1)	—
	368 (1)	—	—	1155 (3)	872 (3)	525 (1)	923 (3)
*MdSFBB11*/*SorbusSFBB9*	**173** (1)	**331** (1)	**240** (1)	**246** (2)	**234** (1)	n.a.	n.a.
	—	—	—	269 (1)	831 (3)	n.a.	n.a.
*SFBB16*	**190** (1)	**190** (1)	**205** (2)	—	—	n.a.	n.a.
	722 (1)	—	391 (1)	361 (2)	221 (1)	n.a.	n.a.
*SFBB17*	—	**115** (2)	—	**181** (2)	—	n.a.	n.a.
	—	—	—	734 (1)	—	n.a.	n.a.

n.a. sequences not reported for *S10-*haplotype^44^.

— sequences not present in the dataset.

() number of sequences in the dataset that show 100% identity with the reported sequences.

Since large size contigs were obtained with Edena assembly (50% of the sequences are larger than 290 bp), we use this to address how many contigs can represent *SFBB* genes. 825 contigs were retrieved from the tblastn of *SFBB3beta* protein (AB270796) and the combined Edena filtered assemblies (identical sequences included within longer sequences have been removed) of the nine anthers transcriptomes. 75 of these contigs present identities higher than 97% with *SLFL*-like genes (not determining GSI specificity)^10^, and thus were also removed. The presence of *SLFL*-like genes in the blast results implies that no other *SFBB* genes are present in these transcriptomes. The remaining 750 sequences could represent *SFBB* genes. The number of contigs per cultivar varied from 57 (‘Northern Spy’) to 99 (‘Red Delicious’ and ‘Honeycrisp’). It should be noted that more than one contig can represent the same *SFBB* allele since the preliminary blast searches revealed that most assembled transcripts are incomplete (see Material and Methods). Indeed 87% of these sequences had a size smaller than 500 bp, and the coding region of the *SFBB* genes is larger than 1Kb. Moreover, if two sequences overlapped but covered different regions, they were both retained at this point. Therefore, to help the assembly and confirm the identified sequences, we characterized *SFBB* sequences from genomic DNA of these individuals, using the primers SFBBgenF and SFBBgenR, described in Aguiar *et al*.^22^, that amplify a region of about 900 bp. Although these primers do not amplify all *SFBB*s^22^, with this additional information most of these sequences will be assembled into larger fragments.

### *M. domestica SFBB* sequences obtained with primers SFBBgenF and SFBBgenR

**Figure 2:**
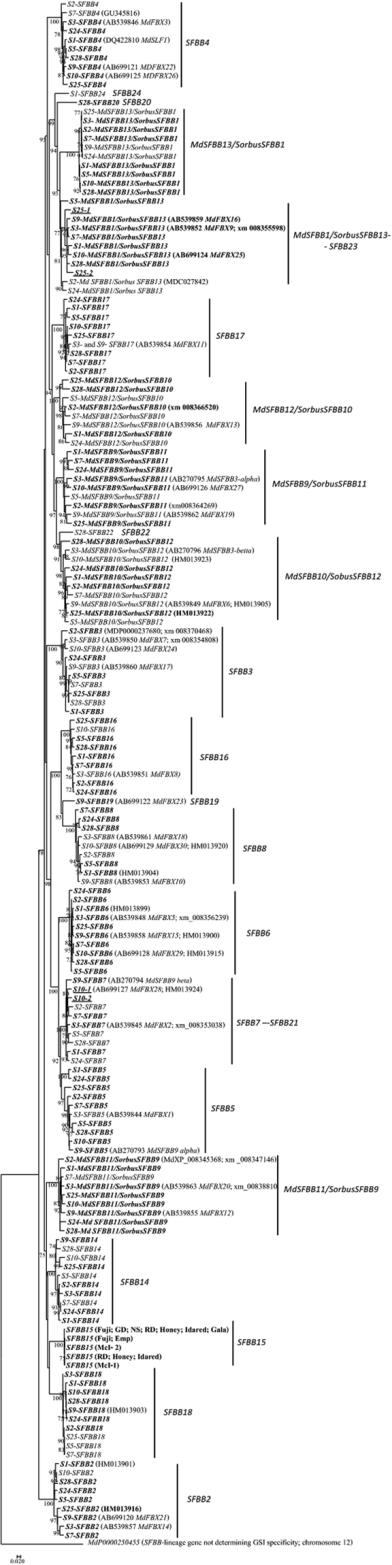
Maximum-likelihood phylogenetic tree showing the relationship of the 173*M. domestica SFBB* sequences obtained for 10 *S*-haplotypes. The tree was rooted with *SFBB* -lineage gene MDP0000250455 (not located in the *S*-locus region, and not involved in GSI)^10^. In brackets are the GenBank acc. numbers for the sequences previously described. In bold are the sequences obtained from the PCR reaction using primers SFBBgenF and SFBBgenR. Numbers below the branches represent bootstrap values above 70.

For each of the nine cultivars an amplification product of about 900 bp was obtained and cloned from genomic DNA with primers SFBBgenF and SFBBgenR^22^. Due to sequence variation within the primer binding sites, these primers are expected to support the amplification of only 65.5% of *Malus* and *Pyrus SFBB* GenBank sequences (n = 165)^22^. Of the 32 *SFBB*s described for *S3*, *S9* and *S10*- haplotypes, 14 of the *Malus* sequences described in the literature^23,27,44^ (Fig. 1 - boxes with vertical lines) could not be amplified for this reason. Sequencing of the insert of more than 30 colonies exhibiting different RFLP patterns for each cultivar (see Material and Methods), revealed 188 coding sequences, plus seven putative pseudogenes (Supplementary Table S5). The presence of identical sequences in two cultivars having a common *S-*haplotype, that are not present in the other cultivars, implies that the sequence comes from the shared *S-*haplotype. It should be noted that, no (or little) diversity is observed at the alleles of the *S*-genes within the same specificity^51–55^. Thus, for all *SFBB* sequences, except those of *SFBB15* (identical sequences are found in cultivars not sharing a *S*-haplotype; boxes with horizontal lines in Fig. 1; Supplementary Table S5), we could assign the sequences into a *S-*haplotype. The putative pseudogene sequences belong to the *S2*-, *S10-*, and *S28-*haplotypes (Supplementary Table S5). One of these sequences corresponds to *S2-SFBB2* gene that presents a nucleotide insertion that is absent in all other *SFBB2* sequences from the other *S*-haplotypes (the star in Fig. 1), that creates in-frame stop codons. This insertion is not a sequencing error since an identical sequence has been obtained from ‘Golden Delicious’ and ‘Honeycrisp’ cultivars, and is also present in the *Malus* genome (NW_007545880.1–1139053… 1137851). All the remaining sequences appeared to be functional *SFBB* alleles. Since most of the *S-*haplotypes are common between cultivars, the number of different coding sequences was 127. Phylogenetic analyses of the coding sequences defined 19 *SFBB* genes (white boxes in Fig. 1; Supplementary Table S5; Fig. 2 (sequences in bold)). Nevertheless, the presence of two sequences for *S10-*haplotype clustering with sequences from other *S*-haplotypes assigned as *SFBB7*, and two sequences for *S25*-haplotype clustering with sequences from other *S*-haplotypes assigned as *MdSFBB1*/*SorbusSFBB13*, implies the presence of 21 *SFBB* genes (Fig. 1; Supplementary Table S5; Fig. 2). It should be noted that 14 of the 32 *SFBB*s described for *S3*-, *S9*-, and *S10*-haplotypes (boxes with vertical lines in Fig. 1; those underlined in Supplementary Table S5)^23,27,44^ were not characterized using the PCR approach, with primers SFBBgenF and SFBBgenR. The 141 different coding sequences, that include the previously reported sequences (using local Blastn, 100% identity and a minimum size for the alignment of 100 bp; see Material and Methods) cover 555 contigs from the Edena assemblies. These were used to enlarge the size of the region sequenced when showing 100% identity in an overlapping region larger than 100 bp.

### Amplification of *MdSFBB1*/*SorbusSFBB13*- *SFBB4*, *SFBB7-SFBB14*, *SFBB16*, and *SFBB18* genes using specific primers

Since the 10 *S*-haplotypes have not been characterized for all *SFBB* genes using SFBBgenF and SFBBgenR primers (Table 2), the 195 Edena contigs that did not show a 100% match to known *SFBB* sequences, may represent uncharacterized alleles and/or new genes. Since polymorphism levels at *SFBB* genes are below 10%^22^, we have used the longest sequences of each gene to identify (using local blast and an overlap of at least 50 bp) putative allelic sequences for each *SFBB* gene. Thus, we inferred that 145 Edena contigs can represent allele sequences of the known genes. Therefore, we used specific primers for *MdSFBB1*/*SorbusSFBB13-SFBB4*, *SFBB7-SFBB14*, *SFBB16*, and *SFBB18* genes (Supplementary Table S6), to amplify the uncharacterized alleles from genomic DNA of the cultivars having that *S*-haplotype. All expected amplification products were cloned and sequenced, as described in Material and Methods. A total of 31 sequences were obtained that included alleles missing for *S2-, S24-MdSFBB1*/*Sorbus SFBB13*, *S10-SFBB2*, *S3-, S7-, S28-SFBB3*, *S2-, S7-SFBB4*, *S2-, S5-, S28- SFBB7*, *S2-SFBB8, S5- MdSFBB9*/*SorbusSFBB11*,

**Table 2:** Alleles for 14 *SFBB* genes that were not identified with SFBBgen primers, but were identified with specific primers.

Gene	Alleles not amplified with SFBBgen primers	Alleles characterized with specific primers
*MdSFBB1*/*SorbusSFBB13*	*S2; S24*	*S2; S24*
*SFBB2*	*S10*	*S10*
*SFBB3*	*S5; S7; S28*	*S5; S7; S28*
*SFBB4*	*S2; S7*	*S2; S7*
*SFBB7*	*S2; S5; S25; S28*	*S2; S5, S28*
*SFBB8*	*S2; S25*	*S2*
*MdSFBB9*/*SorbusSFBB11*	*S5; S28*	*S5*
*MdSFBB10*/*SorbusSFBB12*	*S5, S7, S10*	*S5, S7, S10*
*MdSFBB11*/*SorbusSFBB9*	*S5; S7*	*S7*
*MdSFBB12*/*SorbusSFBB10*	*S3; S5; S7; S24*	*S5; S7; S24*
*MdSFBB13*/*SorbusSFBB1*	*S9; S24; S25*	*S9; S24; S25*
*SFBB14*	*S5; S7; S10; S28*	*S5; S7; S10; S28*
*SFBB16*	*S9; S10*	*S10*
*SFBB18*	*S5; S7; S25*	*S5; S7; S25*

The alleles were named with the *SFBB* and the haplotype from which it was identified.

*S5-, S7-, S10-MdSFBB10*/*SorbusSFBB12*, *S7- MdSFBB11*/*SorbusSFBB9, S5-*, *S7-*, *S24-MdSFBB12*/*SorbusSFBB10, S9-*, *S24-*, *S25-*Md*SFBB13*/*SorbusSFBB1*, *S5-*, *S7-*, *S10-, S28-SFBB14*, *S10-SFBB16*, and *S5-*, *S7-*, *S25-SFBB18* genes (grey blocks in Fig. 1). These sequences were only present in the transcriptome of the cultivars presenting those *S*-haplotypes, when blastn was performed. There were still seven alleles (*S25-SFBB7*, *S25-SFBB8, S28- MdSFBB9*/*SorbusSFBB11*, *S5-MdSFBB11*/*SorbusSFBB9*, *S3-MdSFBB12*/*SorbusSFBB10*, *S10-MdSFBB12*/*SorbusSFBB10*, and *S9-SFBB16*; Fig. 1) that were not amplified using specific primers. They may represent divergent alleles or missing genes in these *S*-haplotypes.

The 157 sequences obtained by PCR, plus the 13 from *S3*-, *S9*-, and *S10*-haplotypes (Fig. 1) show 100% match to 728 Edena contigs. Manual inspection of the 22 remaining Edena contigs revealed eight (from ‘Empire’ and ‘Red Delicious’ transcriptomes) that were assembled into a single larger sequence that shared less than 92% identity with sequences in our dataset. This sequence was present in the ‘Empire’ and ‘Red Delicious’ transcriptome, and was named *S28-SFBB22* (black boxes in Fig. 1). Three other contigs from ‘McIntosh’ were also assembled into a larger sequence that shared 98% identity with *MdSFBB1*/*SorbusSFBB13* sequences. This sequence was only present in the ‘McIntosh’ transcriptome and was called *S25-SFBB23* (black boxes in Fig. 1). Four other sequences from the ‘Fuji’ and ‘Northern Spy’ anthers transcriptomes show overlap and, thus they can be assembled into a larger sequence that shows less than 90% homology with sequences in our dataset. This sequence is only present in transcriptomes of these two cultivars and was called *S1-SFBB24* (black boxes in Fig. 1). These sequences have been confirmed using specific primers, in PCR reactions using genomic DNA of these cultivars. The remaining seven sequences represent almost exclusively 5′and 3′ regions of alleles for which data has been obtained. In conclusion, 173 sequences were obtained that covered more than 80% of the *SFBB* coding region, and 98% of these sequences include the F-box region (60% have the start codon).

### Number of *SFBB* genes in *M. domestica*

The phylogenetic relationship of the 173 sequences obtained in this work support the existence of, at least, 24 *SFBBs* (Fig. 2). *S9-SFBB19* clusters within *SFBB8* and *SFBB16* sequences. Although this sequence could represent a very divergent *SFBB16* allele for the *S9*-haplotype, diversity levels (0.228, after Jukes and Cantor correction) support that this sequence represents a different gene. When primers were designed for this gene sequence (*SFBB19*, Supplementary Table S6), an amplification product with expected size (810 bp) was observed only in ‘Fuji’ and ‘Red Delicious’, the cultivars with the *S9*-haplotype. A similar result was obtained when SFBB19F primer was combined with the SFBBgenR, and SFBB19R primer with SFBBgenF (Supplementary Table S6).

The sequences assigned as alleles of a *SFBB* gene cluster together with strong support. The exceptions are the *SFBB5* gene (*S1-* and *S24-SFBB5* are divergent alleles) and *MdSFBB1*/*Sorbus SFBB13* gene (*S5*- *MdSFBB1*/*Sorbus SFBB13* is a divergent allele). According to the levels of synonymous diversity (0.1 and 0.09 after Jukes and Cantor correction, respectively), there is no support for these sequences representing new genes.

Differences in number and order of *SFBBs* between *S*-haplotypes has been previously observed^23,44^. The number of *SFBB* genes varied from 17 (*S3*-, *S5*-, and *S25-*haplotypes) to 19 (*S1-*, and *S28*-haplotypes) (Fig. 1). When the 24 genes were used as query in a blast search against reads form style, stigma, ovary, filaments, receptacle, petals, sepals, receptacle and young leaves from ‘Golden Delicious’ cultivar transcriptomes, no reads supported the existence of these sequences. In contrast, all *SFBB* genes are expressed in anthers (Supplementary Fig. S3). Therefore, all these genes are expressed in anthers and pollen only, as those involved in GSI (Aguiar *et al*.^10^, and references therein). Except for *SFBB15*, in every case each allele could be associated to a *S*-haplotype, thus indicating linkage to the *S-RNase* gene.

### Associations between *SFBB* genes and the *S-RNase* gene

Progeny segregation from three crosses were analysed to test the linkage between the *S-RNase* and each of the *SFBB-* like genes: ‘Golden Delicious’ (*S2, S3*) × ‘Red Delicious’ (*S9, S28*) - 27 individuals analyzed, ‘Gala’ (*S2, S5*) × ‘McIntosh’ (*S10, S25*) - 48 individuals analyzed, and ‘Fuji’ (*S1, S9*) × ‘Honeycrisp’ (*S2, S24*) - 34 individuals analyzed (Supplementary Table S7; Supplementary Table S8). We used specific primers for conserved regions of each *SFBB* gene (Supplementary Table S6) and the amplification products for each individual was digested with selected enzymes that distinguished the alleles present in each individual, according to the sequences previously obtained (Supplementary Table S7; Supplementary Table S8). This methodology differentiated 17 genes (Supplementary Table S7; Supplementary Table S8) but not *MdSFBB13*/*SorbusSFBB1*, *SFBB15*, and *SFBB18*. These three *SFBB* genes have levels of synonymous diversity bellow 0.013, and thus there were no polymorphic restriction enzyme cut sites that could be used as allele specific markers. The *S-RNase* alleles were also genotyped for the 109 individuals (Supplementary Table S7; Supplementary Table S8), using specific primers (Supplementary Table S9). All 17 *SFBB* genes analyzed were linked with the *S-RNase* gene (Supplementary Table S7; Supplementary Table S8). This result supports the role of these *SFBB*s as *S*-pollen genes.

### Inferring recombination, mutation and diversifying selection at *SFBB* genes

**Figure 3:**
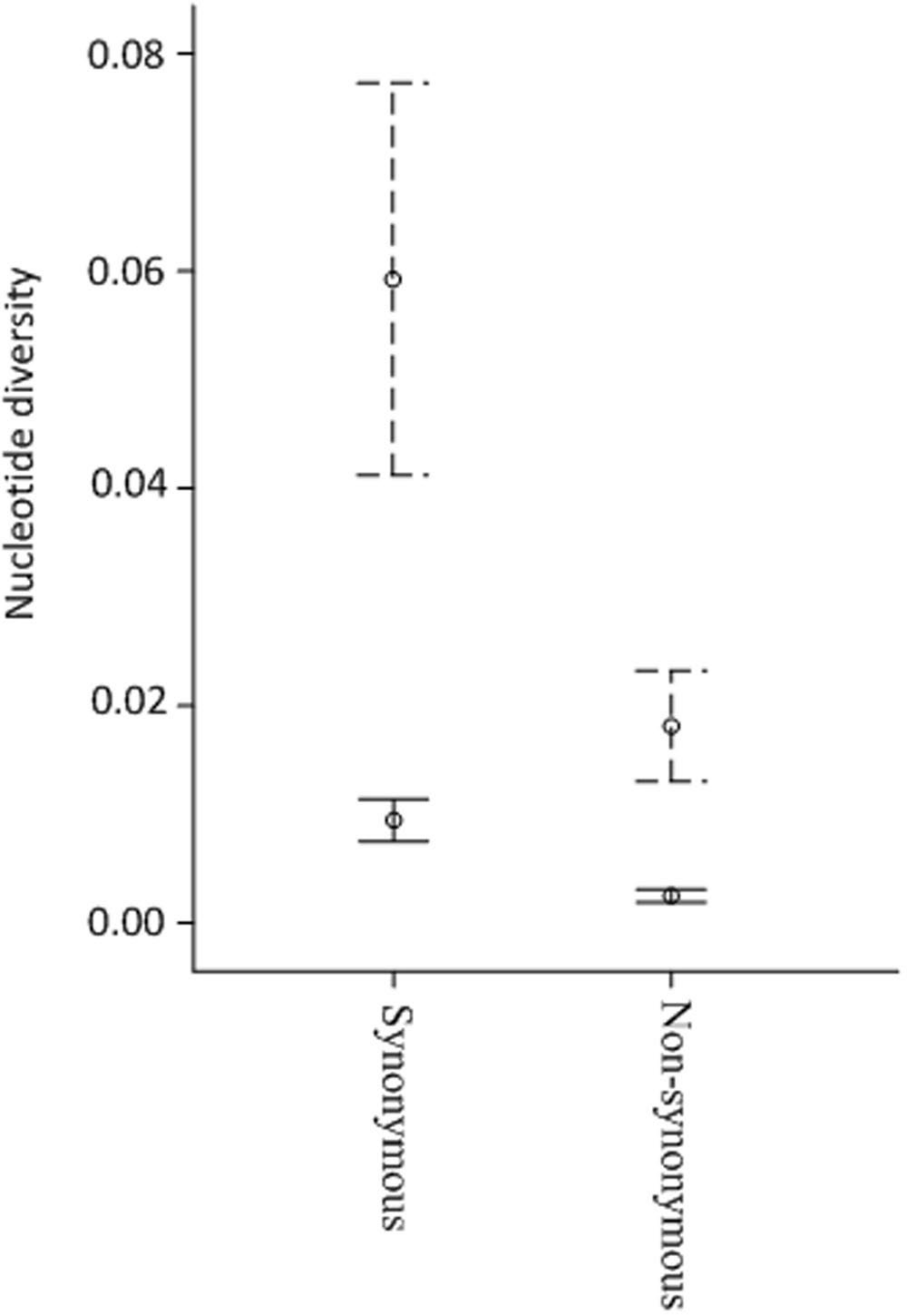
Box plot of synonymous and non-synonymous nucleotide diversity at genes expressed in anthers that are not located at the *S*-locus and *SFBBs* (dotted lines).

Levels of polymorphism for the *SFBB* genes are, on average, 4.2 times lower than those observed for the *S-RNase* (Table 3)^22,23,26,34,48,49^, despite the evidence for specific associations between *SFBBs* and the *S-RNase*. To address the effect of the *S*-locus on the polymorphism levels at *SFBB* genes, the levels of diversity were determined for 126 single copy genes expressed in *M. domestica* anthers transcriptomes (Material and Methods), for which a sequence fragment larger than 100 bp had been obtained in, at least, four cultivars. Both synonymous and non-synonymous diversity levels at the *SFBB*s were higher than those of the 126 *M. domestica* single copy genes expressed in anthers (Fig. 3; Mann-Whitney, P < 0.001). Therefore, *SFBB* diversity is being affected by recombination, and/or diversifying selection. We found evidence for recombination for all *SFBB*s present in more than one *S*-haplotype, except *SFBB15* using different methodologies (Table 3), although for *MdSFBB13*/*SorbusSFBB1*, *SFBB16*, and *SFBB18* genes not all tests support evidence for recombination. It should be noted that RDP uses phylogenetic incongruence, and thus depends on the amount of diversity in the data, and thus is less powerful^56^. Nevertheless, we find evidence for recombination at the *S-RNase* gene. Therefore, the differences observed seem not to be due to recombination alone. On the other hand, we found evidence for diversifying selection only at one, two, and three amino acid positions at *SFBB7*, *SFBB6*, and *SFBB8*, respectively (Table 3). Thus, there is little evidence for diversifying selection at the *SFBB* genes, in contrast with the *S-RNase*. The different selection regimes at the *S*-pollen and *S-RNase* genes seem to be the major cause for the differences on levels of diversity.

**Table 3:** DNA sequence variation summary for sequences of 18 *SFBBs* and the *S-RNase* from *M. domestica*.

Gene	N	*K*_*s*_	*K*_*a*_	Number of sites analysed	Rm	4GT	RDP	Number of synonymous mutations inferred in the phylogeny	Model	Recombination events per synonymous mutation
*MdSFBB1*/*SorbusSFBB13*	9	0.08361	0.03399	898	7	103/6670	3	70.70163	M0	0.042432
*SFBB2*	9	0.05807	0.01914	750	4	12/2926	1	40.32852	M0	0.024796
*SFBB3*	10	0.04639	0.02032	812	3	96/2346	3	33.565	M0	0.089379
*SFBB4*	10	0.06119	0.02836	1156	13	193/10585	1	84.20016	M0	0.011876
*SFBB5*	8	0.08712	0.02078	879	6	82/4371	3	63.89838	M0	0.04695
*SFBB6*	10	0.03746	0.00726	935	7	73/946	2	39.35508	M2 (100; 271)	0.050819
*SFBB7*	8	0.07138	0.02701	770	7	56/3486	1	49.29736	M2 (144)	0.020285
*SFBB8*	9	0.02204	0.0183	809	8	84/1176	1	16.94118	M2 (117;182;304)	0.059028
*MdSFBB9*/*SorbusSFBB11*	9	0.10253	0.03253	837	9	70/8385	3	82.37295	M0	0.03642
*MdSFBB10*/*SorbusSFBB12*	9	0.10285	0.02224	851	8	50/4753	1	79.27522	M0	0.012614
*MdSFBB11*/*SorbusSFBB9*	9	0.07466	0.02444	761	6	31/4186	1	54.92304	M0	0.018207
*MdSFBB12*/*SorbusSFBB10*	8	0.15013	0.03066	465	6	65/2415	2	60.88446	M0	0.032849
*MdSFBB13*/*SorbusSFBB1*	10	0.01162	0.00251	765	1	4/55	0	7.31024	M0	0
*SFBB14*	10	0.07694	0.01797	712	4	19/2485	1	46.15336	M0	0.021667
*SFBB15*	5	0.00212	0.00204	876	0	0/6	0	1.06967	M0	0
*SFBB16*	9	0.05735	0.01659	803	5	34/2556	0	38.2542	M0	0
*SFBB17*	9	0.03279	0.01341	812	1	13/1378	4	25.50332	M0	0.156842
*SFBB18*	10	0.01231	0.0022	543	1	1/10	0	4.15359	M0	0
*S-RNase*	10	0.25541	0.20965	420	26	610/10731	5	108.769	M2 (17)	0.045969
	19*	0.22702	0.19136	660	51	1580/20706	14	220.86	M2 (26)	0.0634

N- number of sequences used.

*K*_*s*_ - ratio of synonymous substitutions per synonymous site.

*K*_*a -*_ ratio of non-synonymous substitutions per non-synonymous site.

Rm- minimum number of recombination events^87^.

4GT - number of pairwise comparisons presenting the four gametic types over the total number of all pairwise comparisons.

RDP- number of independent recombination events^85^.

Model- Yang’s^57^ model used to infer the total number of synonymous mutations implied by the data. In brackets- amino acid sites identified as positively selected, using the method of Yang^57^ implemented in ADOPS^86^ with a probability higher than 90% in both NEB (naive empirical Bayes) and BEB (Bayes empirical Bayes).

*only complete sequences were used.

### Positively selected amino acid sites in 17–19 *M. domestica SFBB* genes at each of the 10 *S*-haplotypes

**Figure 4:**
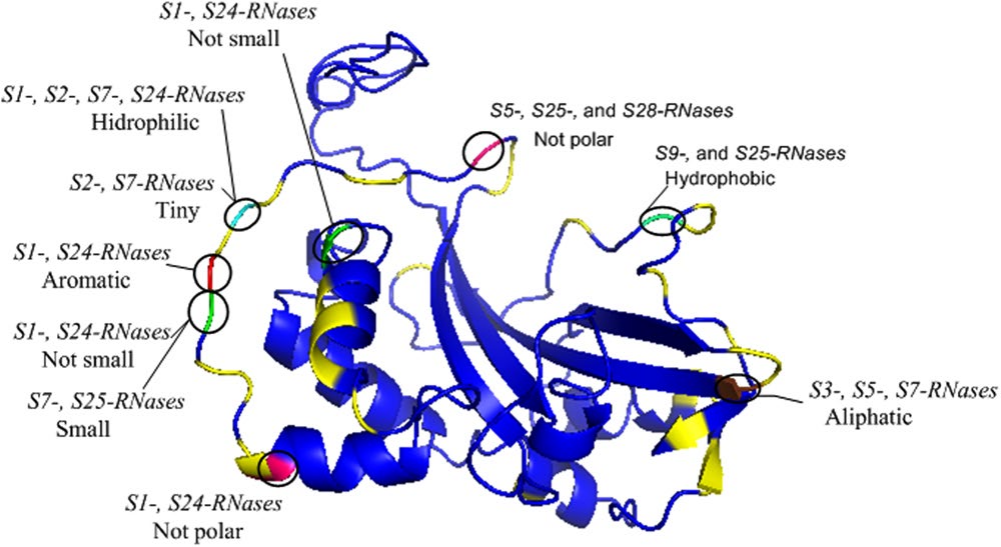
Positively selected amino acid sites mapped onto the S-RNase crystal structure of *M. domestica* S7-RNase, obtained as in Vieira *et al*.^61^. Positively selected amino acid positions that are putatively involved in SFBB specificities recognition are highlighted. The features of those amino acid positions that have been inferred to be important for discriminating different SFBBs are shown. Green- size, brown- aliphatic, red- aromatic, pink- polarity, light green- hidrophobicity, and light blue- hidrophobicity and size.

In the collaborative non-self -recognition model each *S*-pollen gene recognises a sub-set of non-self- S-RNases, but not the S-RNase of its *S-*haplotype^19–24^. Recently, Kubo and co-authors^20^ proposed for *Petunia* species a more detailed model that falls under the general collaborative non-self -recognition model. Under Kubo and co-authors^20^ model, having either a diverged or deleted allele at a *SLF* gene, whose product usually recognizes a given Sx-RNase, is the way by which recognition avoidance of the own Sx-RNase is achieved. All non-divergent alleles would recognize the Sx-RNase. In agreement with this model, in *Petunia*, phylogenetic analyses show divergent and non-divergent alleles as two distinct allele groups. The phylogenetic inferences led to the identification of *SLF* genes that recognize seven S-RNases, among eight *S*-haplotypes analysed, and in five cases their predictions have been confirmed with experimental evidence. It should, however, be noted, that in *Petunia*, different non-divergent alleles of the same *SLF* gene (see Fig. 4 in ^34^for *SLF1* gene) can recognize different S-RNases. For instance, *Petunia S7*- and *S5*-*SLF1* alleles can recognize S17- and S9-RNases, but S11-SLF1 only recognizes the S17-RNase, and not the S9-RNase.

In *Malus*, for six *SFBB* genes, alleles could be found for only 8 or 9 of the 10 *S*-haplotypes analysed (missing boxes in Fig. 1). This finding could suggest that the *Malus* system may work in a way similar to that proposed by Kubo and co-authors^20^ for *Petunia*, since it is conceivable that if more *S*-haplotypes are analysed missing alleles will be found at all *SFBB* genes. In *Malus*, no divergent alleles were, however, found at *SFBB* genes. It should be noted that in *Petunia*, divergent alleles show less than 90% identity with non-divergent alleles, and in *Malus* such value is only observed when different genes are being compared. This could be due to the effect of recombination, since in *Petunia* intragenic recombination is only inferred for two *SLF* genes^20^ and in *Malus* recombination is inferred for 94% of the *SFBB* genes (Table 3). In the presence of recombination it is not possible to have clearly defined allele groups. Nevertheless, we can still make some inferences, by assuming that for any *SFBB* gene, alleles that are identical at the amino acid sites responsible for specificity recognition, are targeting the same set of S-RNases. Moreover, those alleles cannot recognize the S-RNases to which they are linked. Indeed, polymorphism levels at the *SFBB* genes are low (see above), and thus, natural selection must favour diversification of *SFBB* genes within a *S*-haplotype^22,34^. Evidence for adaptive evolution at the *SFBB* paralogous genes has been found using codeML^57^ and 11 *SFBB* genes of two *Sorbus S*-haplotypes^22^. 12 amino acid sites were identified as being positively selected, and these amino acid positions were found to be polymorphic when comparing the alleles of different *S*-haplotypes for each *SFBB* gene, thus supporting the involvement of these amino acids in specificity determination^22^. Using the same methodology and the 17 to 19*M. domestica SFBB* genes of each *S*-haplotype here characterized, we identified 21amino acid sites under positive selection (Table 4; B + database^58^ (bpositive.i3s.up.pt; see the *Malus* SFBB BP2017000011 dataset)). Supplementary Fig. S4 shows these amino acid sites on top of a reference alignment of the *SFBB4* gene. Since most of the sequences here used do not cover the region where positively selected amino acid position 377 is located, this position has not been considered in the remaining analyses. Assuming that the positively selected amino acid sites are those determining *S*-pollen specificity, the *MdSFBB13*/*SorbusSFBB1* and *SFBB18* genes, which do not present polymorphism at these positions, are not involved in the recognition of any of the 10 *S-RNases* here studied. Assuming that within a *SFBB* gene an allele showing one difference at the positively selected amino acid positions (those that are involved in specificity determination) is sufficient to prevent the recognition of a given *S-RNase* specificity, the number of sequences that can be distinguished based on these amino acid positions only, gives insight into the maximum number of different *S-RNase* specificities recognized by a single *SFBB*. In our dataset, the maximum number of different *S-RNase* specificities recognized by a single *SFBB* gene is eight (see *MdSFBB1*/Sorbus*SFBB13* gene; Table 4). Positively selected amino acid sites that are invariant within a given *SFBB* gene can be devoted to the recognition of the same S-RNase as proposed by Kubo and co-authors^20^. In the case of *SFBB* genes presenting missing alleles, the S-RNase that is recognised is likely the one linked to the *S*-haplotype presenting the missing allele, as proposed by Kubo and co-authors^20^. Within a *S-*haplotype there is always a minimum set of genes that can, in principle, recognize all *S-RNase* specificities here considered, but the self *S-RNase* (for instance for the *S1*-haplotype, genes *MdSFBB1*/*SorbusSFBB13*, *SFBB3*, *SFBB7*, *MdSFBB11*/*SorbusSFBB9*, could alone recognize the *S2*-, *S3*-, *S5-*, *S7-, S9-*, *S10-*, *S24, S25*, and *S28-RNases*; Table 4). For the *S-*haplotypes here considered, on average, there are five (3 to 7) *SFBB* genes that alone can recognise all the *S-RNase* specificities here considered but the self *S-RNase* (Table 4). Thus, there are multiple *SFBB* genes recognizing the same *S-RNase* specificity.

**Table 4:** Amino acid composition for each SFBB in the 10 *S*-haplotypes for the amino acid sites identified as positively selected in intra-haplotypic analyses of *M. domestica* SFBBs using 10 *S*-haplotypes (see B + database^58^ (bpositive.i3s.up.pt; see the *Malus* SFBB BP2017000011 dataset, for analyses).

Gene	71	77	81	112	117	119	132	160	162	169	170	188	217	232	235	251	253	281	303	304	*S*-haplotype
*MdSFBB1*/*SorbusSFBB13*	A	N	P	L	F	Q	A	P	K	I	G	Q	M	E	H	N	G	E	D	E	*S*1
	.	.	.	.	.	E	.	.	.	.	.	.	.	.	.	T	.	.	.	A	*S*2
	.	.	.	.	.	.	.	.	Q	.	.	.	.	.	.	I	.	.	.	.	*S*3
	.	.	.	.	.	E	.	.	.	.	.	.	.	D	.	T	.	.	.	A	*S*5
	.	.	.	.	.	.	.	.	.	.	.	.	.	.	.	T	.	.	.	.	*S*7, *S*25
	.	.	.	.	.	.	.	.	T	.	.	.	.	.	.	I	.	.	.	A	*S*9
	.	.	.	.	.	.	.	.	.	.	.	.	.	.	.	I	.	.	.	.	*S*10, *S*28
	.	.	.	.	.	E	.	.	.	.	.	.	T	.	.	T	.	.	.	.	*S*24
*SFBB2*	M	R	R	L	H	S	V	P	E	T	Q	K	T	D	N	S	D	L	D	N	*S*1, *S*9, *S*24, *S*28
	.	.	.	.	L	.	.	.	K	S	.	.	.	.	.	.	.	.	.	.	*S*3
	.	.	.	.	N	.	.	.	.	.	.	.	.	.	.	.	G	.	.	.	*S*5
	.	.	.	.	.	.	.	Q	G	.	.	.	.	.	.	.	.	.	.	.	*S*7
	.	.	.	.	.	.	.	.	.	.	.	.	.	.	.	.	G	.	.	.	*S*10
	.	.	.	.	.	.	.	.	.	.	.	.	M	.	.	.	.	.	.	.	*S25*
*SFBB3*	F	Q	R	R	H	Q	E	P	E	T	H	Q	T	S	P	T	G	N	E	D	*S*1
	.	.	H	.	.	H	.	.	.	.	.	.	.	T	.	R	.	K	.	.	*S*2
	.	.	.	.	P	.	.	.	.	.	.	.	.	T	.	.	.	E	.	.	*S*3
	.	.	.	.	P	.	.	.	.	.	.	.	.	.	.	.	.	.	.	.	*S*5, *S*7, S9, *S*24*, S*25*, S*28
	.	.	.	.	P	E	.	.	.	.	.	.	.	T	.	I	.	E	.	.	*S*10
*SFBB4*	V	K	H	R	H	L	S	L	G	D	G	R	M	K	P	R	G	E	Q	D	*S*1, *S*24
	.	.	.	.	.	I	.	.	.	.	.	.	.	.	.	.	.	.	E	.	*S*2
	.	.	.	.	.	.	.	.	.	.	.	.	.	.	.	.	.	.	E	.	*S*3, *S*5, *S*9, *S*10
	.	.	Q	.	.	.	.	.	.	.	.	.	.	.	.	.	.	.	E	.	*S*7
	.	.	.	.	.	.	.	.	S	.	.	.	.	.	.	.	.	.	E	.	*S*25
	.	.	.	.	.	.	L	.	.	.	.	.	.	.	.	.	.	.	E	.	*S*28
*SFBB5*	V	R	Q	M	N	E	V	—	K	I	K	Q	M	D	P	N	N	K	—	—	*S*1, *S*24
	.	.	.	.	.	.	.	.	.	V	.	R	.	.	.	M	.	.	—	—	*S*2, *S*3, *S*5, *S*7, *S28*
	.	.	.	.	.	.	.	.	.	V	.	R	.	.	.	C	.	.	—	—	*S*9
	.	.	.	.	.	.	.	.	.	.	.	R	.	.	.	.	.	.	—	—	*S*10
	.	K	.	.	.	D	.	.	.	.	.	R	.	.	.	M	.	.	—	—	*S*25
*SFBB6*	V	R	R	I	N	Q	V	—	M	L	K	R	T	D	P	N	N	K	—	—	*S*1, *S*25
	.	.	.	.	.	.	.	.	T	.	.	.	.	.	.	.	.	.	.	.	*S*2, *S*3, *S*5, *S*7, *S*9, *S*10, *S*24, *S*28
*SFBB7*	V	R	Q	I	N	E	.	—	K	T	K	R	T	E	P	Y	N	N	.	.	*S*1
	.	.	.	M	.	.	V	.	.	I	.	.	.	.	.	.	.	K	.	.	*S*2
	.	.	.	M	.	.	V	.	.	.	.	.	.	.	.	.	.	K	.	.	*S*3, *S*5, *S*7
	.	.	.	M	.	.	V	.	.	.	.	.	M	.	.	.	.	K	.	.	*S9*
	.	.	.	M	.	.	.		.	.	.	.	.	.	.	.	.	K	—	.	*S*10—1
	.	.	.	R	.	.	V		.	.	.	.	.	.	.	.	.	K	—	.	*S*10—2
	.	.	.	.	.	.	V		.	.	.	.	.	.	.	.	.	K	.	.	*S*24
	.	.	.	M	.	D	.		.	.	K	.	.	.	.	.	.	K	.	.	*S*28
*SFBB8*	A	E	Q	R	E	E	V	G	K	T	K	R	M	K	P	C	V	K	—	—	*S*1, *S*5
	.	.	.	K	.	.	.	.	.	.	.	.	.	.	.	.	.	.	.	.	*S*2, *S*9, *S*10, *S*24, *S*28
	.	Q	.	K	.	.	.	.	.	.	.	.	.	.	.	.	L	.	.	.	*S*3
	.	Q	.	K	.	.	.	.	.	.	.	.	.	.	.	.	.	.	.	.	*S*7
*MdSFBB9*/*SorbusSFBB11*	A	Q	Q	L	F	L	A	P	E	S	Q	R	T	T	S	T	G	R	E	D	*S*1, *S*2, *S*7, *S*24
	.	.	.	.	.	.	.	.	.	N	.	.	.	.	.	.	.	.	.	.	*S*3
	P	.	.	.	.	.	.	L	.	.	.	.	.	.	.	.	.	.	.	.	*S*5
	.	.	.		.	.	.	.	.	.	.	.	A	.	.	.	.	.	.	.	*S9*
	.	.	.	.	.	.	.	.	.	N			M	.	.	.	.	.	.	.	*S10*
	.	.	.	.	.	.	.	.	.	.	.	.	A	A	.	.	.	.	.	.	*S*25
*MdSFBB10*/*SorbusSFBB12*	A	Q	Q	L	F	L	A	P	K	T	Q	R	T	Q	H	S	S	T	E	D	*S*1, *S*5, *S*25
	.	.	.	.	.	.	V	.	.	.	.	.	.	.	.	.	N	.	.	.	*S*2, *S*7
	.	.	.	.	.	.	.	.	.	.	.	.	.	K	.	.	N	.	.	.	*S*3
	.	.	.	.	.	.	.	.	.	.	.	Q	.	.	.	.	.	.	.	.	*S*9
	.	.	.	.	.	.	.	.	.	N	.	.	.	S	.	.	N	.	.	.	*S*10
	.	.	.	.	.	.	.	.	.	.	.	.	M	.	.	.	.	.	.	.	*S24*
	.	.	.	.	.	.	.	.	.	.	.	.	.	E	.	.	.	.	.	.	*S*28
*MdSFBB11*/*SorbusSFBB9*	M	Q	Y	T	P	Q	I	P	E	I	E	Q	T	K	Q	N	G	K	E	D	*S*1
	.	.	.	.	.	.	T	.	.	.	.	.	.	.	.	S	.	.	.	.	*S*2
	.	.	.	.	.	.	T	.	.	.	.	.	A	.	.	.	.	.	.	.	*S*3
	.	.	.	.	.	.	T	.	.	.	.	.	.	.	.	.	.	.	.	.	*S*7, *S*10, *S*24
	.	.	.	M	.	.	T	.	.	.	.	.	.	.	.	.	.	.	.	.	*S*9, *S*28
	.	.	.	.	.	.	T	.	Q	.	.	.	.	.	.	.	.	.	.	.	*S*25
*MdSFBB12*/*SorbusSFBB10*	P	K	Q	L	F	Q	V	E	G	T	E	Q	T	T	S	T	D	T	G	D	*S*1, *S*9
	—	—	—	—	—	—	A	.	.	.	.	.	.	.	.	.	.	.	.	.	*S*5
	.	.	.	.	.	.	.	.	.	.	.	.	M	.	.	.	.	.	.	.	*S2, S*7
	—	—	—	—	—	.	.		.	.	.	.	.	.	.	.	.	—	—	—	*S*24
	.	Q	.	.	.	.	.	.	.	.	.	.	.	K	.	.	.	.	.	.	*S*25
	.	Q	.	.	.	.	L	.	.	.	.	.	.	N	.	.	.	.	.	.	*S*28
*MdSFBB13*/*SorbusSFBB1*	N	R	P	L	F	E	A	S	R	I	T	Q	T	E	C	T	E	K	D	E	*S*1, *S*2, *S*3, *S*5, *S*7, *S*9, *S*10, *S*24, *S*25, *S*28
*SFBB14*	M	K	Y	L	P	Q	A	P	E	I	G	Q	I	K	P	S	G	K	E	D	*S1, S2*, *S*3, *S*5, *S*7, *S24*
	.	.	.	.	.	.	.	.	.	.	.	.	.	.	.	.	.	.	D	.	*S*9, *S*25
	.	.	.	.	.	.	.	.	.	S	.	.	.	E	.	.	.	.	.	.	*S*10
	.	.	.	.	.	.	.	.	.	.	R	.	.	.	.	.	.	.	.	—	*S*28
*SFBB16*	T	D	R	Q	E	I	L	G	K	T	K	R	T	K	P	S	D	K	—	—	*S*1, *S*2, *S*3, *S*7, *S*24
	M	.	.	.	.	.	.	.	.	.	.	.	.	.	.	.	.	.	.	.	*S*5, *S*25, *S*28
	M	.	.	.	.	.	.	.	.	I	.	.	.	.	.	.	.	.	.	.	*S*10
*SFBB17*	T	N	Q	L	Y	L	A	P	K	V	R	Q	T	K	S	T	A	K	D	K	*S*1, *S*2, *S*3, *S*7, *S*9, *S*10, *S*25, *S*28
	.	.	.	.	.	.	.	.	.	.	.	.	.	R	.	.	.	.	.	.	*S*5
	.	.	.	.	.	.	.	.	.	.	.	.	.	E	H	.	G	.	.	.	*S*24
*SFBB18*	M	D	Y	M	P	L	T	P	E	T	R	R	T	K	P	T	G	K	E	D	*S*1, *S*2, *S*3, *S*5, *S*7, *S*9, *S*10, *S*24, *S*25, *S*28

Sites were identified using the method of Yang^57^ implemented in ADOPS^86^ with a probability higher than 95% in NEB (naive empirical Bayes) or BEB (Bayes empirical Bayes) in at least one *S*—haplotype. The positions are according to the alignment of *SFBB4* gene presented in Supplementary Fig. S4.

It is known that protein-protein interactions depend on properties such as residue interface propensities, hydrophobicity and conformational changes^59,60^. In *P. hybrida* it has been shown that one alteration of an amino acid under positive selection at the C-terminal SLF protein, was sufficient to change *S*- pollen specificity, because it causes a change in the surface electrostatic potential^36^. To identify features at the pistil amino acid sites under positive selection^61^(see Supplementary Fig. S5 for those amino acid sites for the 10 *S-RNases* here analysed), such as hydrophobicity, polarity, aliphatic, charge, size, and aromatic, that can determine pistil-pollen interactions, we determined whether these amino acid proprieties are exclusively found in a group of *S-RNases* that share in their *S*-haplotype, for a particular *SFBB* gene, *SFBB* alleles with identical sequences at the amino acids under positive selection (*SFBB* alleles that recognize the same *S-RNase* specificity; Table 4). For instance, *S1-* and *S24-* haplotypes have identical amino acids at sites under positive selection at two genes, namely *SFBB4* and *SFBB5*. Therefore, none of these *SFBB* genes is able to recognize either the *S1-* and *S24-RNases*. Comparing the above features of the amino acids under positive selection for the *S1-* and *S24-RNases* to the remaining *S-RNases* present in the cultivars analysed, we observed that *S1-* and *S24-RNases* have proprieties that are unique in four of these sites (the two *S-RNases* are the only ones that at amino acid position 80 present an aromatic amino acid, at positions 81 and 125 amino acids are not small, and at position 88 the two *S-RNases* are the only ones that are not polar; Fig. 4). This suggests that the amino acid composition of the *S-RNase* at these sites prevents the interaction with the protein encoded by the *SFBB4* gene at amino acid position 304 (that is unique in the *SFBB4* alleles analysed, Table 4) or/and with *SFBB5* gene at position 188 (Table 4). For *S2- S7-MdSFBB10*/*SorbusSFBB12*/*S2- S7-RNases* and also *S2- S7-MdSFBB12*/*SorbusSFBB10*/*S2- S7-RNases*, at amino acid position 78, *S2-, S7-RNases* are the only ones presenting a tiny amino acid. This position prevents the interaction with the protein encoded by the *MdSFBB10*/*SorbusSFBB12* gene at amino acid position 132 (Table 4), and with *MdSFBB12*/*SorbusSFBB10* gene at position 217 (Table 4). Results pointing to one amino acid site preventing the protein-protein interaction between *S-RNase* and a *SFBB* are also obtained for *S3*-*, S5*-*, S7- SFBB7*/*S3-, S5-, S7-RNases* (at amino acid position 200 *S3-, S5-, S7-RNases* are the only ones presenting an aliphatic amino acid; Fig. 4), *S1-*, *S2-*, *S7-*, *S24- MdSFBB9*/*SorbusSFBB11*/*S1-, S2-, S7-, S24-RNases* (at amino acid position 78 *S1-, S2-, S7-, S24-RNases* are the only ones presenting a non hydrophobic amino acid; Fig. 4), *S7-, S25-MdSFBB1*/*SorbusSFBB13*/*S7-, S25-RNases* (at amino acid position 81 *S7-, S25-RNases* are the only ones presenting a small amino acid; Fig. 4), *S9-S25-SFBB14*/*S9- S25-RNases* (at amino acid position 227, *S9-, S25-RNases* are the only ones presenting a hydrophobic amino acid), and *S5-, S25-, S28-SFBB16*/*S5-, S25-, S28-RNases* (at amino acid position 70 *S5-, S25-, S28-RNases* are the only ones presenting a non polar amino acid; Fig. 4).

## Discussion

The number of *M. domestica SFBB* genes present in a given *S*- haplotype varies from 17 to 19 (Fig. 1). A similar number of genes is observed in *Petunia*^20,21^, although the two systems may have evolved independently^10^. Under the assumption that each *S*-pollen can recognize a different proportion of target *S-RNases* (according to *Petunia* transformation experiments a *S*-pollen gene can recognize 18.6% of S-RNases on average), Monte Carlo simulations revealed that between 16 to 20 *S*-pollen genes are sufficient to recognize 40 *S-RNases* specificities^20^. Since the number of *Malus SFBB* genes is lower than the number of S-RNase specificities described in *M. domestica*^61^, each *S*-pollen must recognize a different proportion of target *S-RNases*, like in *Petunia*. In *M. domestica* there are 59 *S-RNase* unique sequences in GenBank, that according to the sites under positive selection^61^, define 34 *S-RNase* specificities. Moreover, our results show that within a *S-*haplotype, 20% of the genes can recognise all the *S-RNase* specificities studied but the self *S-RNase*. Therefore, it is not surprising that the two systems have a similar number of *S*-pollen genes, independently of their evolution.

In *Petunia*, it has been observed that either divergent or absent alleles at a particular *S*-pollen gene are those determining *S-RNase* specificity recognition^20^. In *M. domestica* we find six genes that are absent in five *S*-haplotypes (*S3*-, *S5*-, *S9*-, *S25*-, and *S28*- haplotypes; Fig. 1; Table 2) and six genes that were detected in a single *S*-haplotypes (*S1*-, *S9*-, *S10*-, *S25*-, and *S28*- haplotypes; Fig. 1 and Fig. 2). Therefore, it seems that absent alleles are also important in *M. domestica* specificity determination, although these observations are not sufficient to account for the 10 *S-RNase* specificities here analysed. When amino acids under positive selection are considered, we can account for all specificities in the data set. Furthermore, the data supports the prediction that different *SFBB* genes are involved in the recognition of the same non-self *S-RNase* specificity.

Although we do not know how *S*-pistil and *S*-pollen proteins interact to allow self/non-self recognition and discrimination, the chemical characteristics of amino acids under positive selection at both proteins must be determinant for such interactions. Under the assumption that two *SFBB* alleles, from two different *S*-haplotypes, showing identical amino acids at sites under positive selection cannot recognize any of the two *S-RNase* specificities of the two *S*-haplotypes, we find at the corresponding *S-RNase* chemical characteristics at amino acids under positive selection such as hydrophobicity, polarity, aromatic, aliphatic, and size, that are exclusively found in these, and thus must be involved in the self/non-self recognition (Fig. 4). The assumption that one amino acid under positive selection is sufficient for self/non-self recognition seems to be realistic since in *Petunia* the alteration of a single C-terminal amino acid under positive selection at one *S*-pollen gene is sufficient to change *S*-pollen specificity^36^. Here we identified putative interactions for the amino acid positions unique in the *SFBB* alleles that could recognize as self a particular set of *S-RNase* specificities (Fig. 4), but further interactions can be predicted by considering amino acid sites under positive selection that are shared with other *SFBB* alleles. Such inferences are essential for guided experimental validation.

Having multiple *SFBB*s to detoxify a given non-self S-RNase will reduce the loss of cross-compatibility caused by mutations and/or recombination^62^. In *M. domestica* we found evidence for duplications within a *S*-haplotype for two genes (within *S10*, for the *SFBB7* vs. *SFBB21* genes, and within *S25*, for the *MdSFBB1*/*SorbusSFBB13* vs. *SFBB23* genes). For the *MdSFBB1*/*SorbusSFBB13* vs. *SFBB23* gene pair, the observed sequence relationships are not those expected under a model of gene duplication without intragenic recombination. Nevertheless, it is compatible with a model where there is intragenic recombination and where the duplicated gene no longer recombines with the gene that gave origin to it, making most alleles of the *MdSFBB1*/*SorbusSFBB13* similar among them, but not the *MdSFBB1*/*SorbusSFBB13* and the *SFBB23* gene. Recombination can also contribute to the gene number variation observed in *S-*haplotypes, as well as in the development of chimeric *SFBB*-genes that can encode novel specificities. Intragenic recombination is detected in all *SFBB* genes showing more than two different sequences, except for *SFBB15*. In *Petunia* evidence for *S-*pollen genes intragenic recombination has been reported^20^. Therefore, duplication and recombination are essential for functional diversification, and thus for generation of *S-*pollen specificities. Nevertheless, it is possible that the number of *SFBB* genes per *S*-haplotype is constrained by the fitness costs of having more genes, as observed for genes involved in the recognition of pathogen avirulence^63–73^.

## Material and Methods

### Plant material and RNA-DNA extractions

In Pyrinae there are no homozygous lines and thus, in this work, we selected a set of nine cultivars [‘Fuji’ (*S1*, *S9*), ‘Northern Spy’ (*S1*, *S3*), ‘Golden Delicious’ (*S2*, *S3*), ‘Gala’ (*S2, S5*), ‘Honeycrisp’ (*S2*, *S24*), ‘Idared’ (*S3*, *S7*), ‘Red Delicious’ (*S9*, *S28*), ‘McIntosh’ (*S10*, *S25*), and ‘Empire’ (*S10*, *S28*)], where two to three cultivars share six specificities (*S1, S2, S3, S9, S10*, and *S28*). Since no or little diversity is expected for alleles of the *S*-genes within the same specificity^51–55^, these are, in principle, equivalent to the use of two biological replicates for the *S*-locus genes. Three of these *S*-haplotypes were used as controls since the *SFBB* genes have already been characterized^23,27,44^. Anthers from flower buds 1–3 days prior to opening were collected from trees of the above nine *M. domestica* cultivars, growing at Michigan State University campus, East Lansing, Michigan. The anthers were immediately frozen in liquid nitrogen and stored at −80 °C for RNA extraction. Anthers were used since *SFBBs* show higher expression levels at this tissue (Fig. 4 in Aguiar *et al*.^10^). Flower buds were also collected from these individuals for DNA extraction. Additional tissues were collected for ‘Golden Delicious’: petals, sepals, filaments, receptacle, styles, stigmas, and ovaries from open flowers, and immature leaves from new shoot growth for RNA extraction.

Controlled crosses between ‘McIntosh’ × ‘Gala’, ‘Fuji’ × ‘Honeycrisp’, and ‘Golden Delicious’ × ‘Red Delicious’ were performed and 48, 34, and 27 seeds, respectively, were obtained. The seeds where germinated and leaves were collected from the seedlings and stored at −20 °C for DNA extraction. No permits were required for the field collection, since the plant location is part of Michigan State University and *M. domestica* is not an endangered or protected species.

### RNA and DNA extraction, RNA Library Construction, and Sequencing

Total RNA was extracted using the mirVana^TM^ miRNA Isolation Kit (Ambion), using the manufacturer’s guidelines for recovery of total RNA. RNA quantity was assessed using a NanoDrop v.1.0 (Thermo Scientific) and RNA quality was evaluated by BioRad’s Experion System. cDNA libraries construction and sequencing was performed using the Illumina TruSeq protocol and reagents with 100-bp, paired-end sequencing. A total of 138380723 read pairs were obtained for the anther transcriptomes (Supplementary Table S1). Genomic DNA was extracted using the method of Ingram *et al*.^74^ or the Puregene® DNA purification system (Gentra Systems, Minneapolis, USA).

### Transcriptome Assembly and Coverage

The Transcriptome Shotgun reads have been deposited at Sequence Read Archive (SRA) under BioProject PRJNA419119. Only high quality reads were used. Before assembly, adaptor sequences were removed from raw reads. FASTQC reports were then generated and based on this information the resulting reads were trimmed at both ends. Nucleotide positions with a score lower than 20 were also masked (replaced by an N). These analyses were performed using the FASTQ tools implemented in the Galaxy platform^75,76^. The total number of reads for each transcriptome is presented in Supplementary Table S1. To assess the changing rate of new gene detection as a function of sequencing sampling for the nine anthers transcriptomes here obtained (Supplementary Fig. S1A), plus the nine Golden delicious tissues analysed (Supplementary Fig. S1B), we have obtained an accumulation curve by dividing the reads in sets of one million paired reads and looking for the number of *M. domestica* CDS, retrieved from the *M. domestica* RefSeq at NCBI, that show evidence for expression. Blastn search using as query the 33 *SFBB* sequences previously identified for *S3*-, *S9*- and *S10*- haplotypes^23,27,44^ and identities higher than 90% revealed 10 *SFBB*s in the *M. domestica* RefSeq (Supplementary Table S2). FPKM values in these 18 transcriptomes were estimated using Express as implemented in Trinity (default parameters)^77^. The reads were then used in the transcriptome assembly using Trinity (default parameters)^77^ and also using Edena^78^ with the following K-mer values 20, 25, 30, 35, 40, 45, 50, 55 and 60. Assembly statistics for both assemblies were obtained with ABySS 2.0^79^ (Supplementary Table S3 and Supplementary Table S4). The resulting files were merged and contigs that have a 100% match along the full sequence with larger contigs were eliminated. All contigs were used as subject for tblastn searches using local blast^80^, and the *SFBB9* (*MdSFBB3-Beta*; AB270796) sequence as query, and an expect value of 0.05. It should be noted that when using such parameters we also obtained sequences that show high identity (more than 97% identity over more than 100 bp) with previously reported *SFBB*-like genes. Therefore, it is unlikely that using this methodology we missed any *SFBB* gene. Nevertheless, not all alleles of each *SFBB* gene were obtained here,when the selected contigs were used as the query to perform a local blastn search^80^, against a database of 33 *SFBB* sequences from *S3-*, *S9*-, and *S10*-haplotypes^23,27,44^ (see Results).

### Amplification of *SFBB* genes

Genomic DNA of each of the nine *M. domestica* cultivars was used as template in PCRs using primers SFBBgenF and SFBBgenR^22^. Standard amplification conditions were 35 cycles of denaturation at 94 °C for 30 seconds, primer annealing at 48 °C for 30 s, and primer extension at 72 °C for 2 min. The amplification products were cloned using the TA cloning kit (Invitrogen, Carlsbad, CA). For each cultivar and amplification product, the insert of an average of 60 colonies was cut separately with *Rsa*I, *Alu*I, *Ava*II and *Sau*3AI restriction enzymes. For each cultivar and restriction pattern two colonies were sequenced. The ABI PRISM BigDye cycle-sequencing kit (Perkin Elmer, Foster City, CA), and specific primers, or the primers for the M13 forward and reverse priming sites of the pCR2.1 vector, were used to prepare the sequencing reactions. Sequencing runs were performed by STABVIDA (Lisboa, Portugal). Local blastn was performed using theses sequences as query and *Sorbus SFBB* genes^22^ as subject. Sequences with homology higher than 95% were grouped as alleles of a particular gene.

Sequences were then aligned, using clustalW as implemented in Mega7^81^ to identify conserved regions in all sequences for a given gene but that are different in other *SFBB* sequences. These regions were used to design specific primers for *SFBB* genes (Supplementary Table S6). These primers were used to amplify *SFBB* alleles from genomic DNA of *M. domestica* cultivars for which allele sequences were not obtained with SFBBgenF and SFBBgenR primers. Amplification conditions are described in Supplementary Table S6. The amplification products were cloned as described above. For each cultivar and amplification product, the insert of an average of 20 colonies was cut separately with *Rsa*I, *Alu*I, *Ava*II and *Sau*3AI restriction enzymes. The colonies that show a different restriction pattern from that of the alleles obtained with SFBBgenF and SFBBgenR primers were selected for sequencing. For each pattern three colonies were sequenced, as described above.

### Genotyping and linkage analyses between 15 *SFBB* genes and nine *S-RNases*

For 17 out of the 18 *SFBB* genes present in more than one *M. domestica* cultivar, we were able to infer the allele that goes with a particular *S-RNase* (see Results). To show that these 17 *SFBB* genes are located in the *S*-locus region, we used 48, 34, and 27 individuals of the crosses ‘McIntosh’ (*S10, S25*) × ‘Gala’ (*S2, S5*), ‘Fuji’ (*S1, S9*) × ‘Honeycrisp’ (*S2, S24*), and ‘Golden Delicious’ (*S2, S3*) × ‘Red Delicious’ (*S9, S28*), respectively, that were genotyped for *S-RNase* alleles, using specific primers (Supplementary Table S9). For *SFBB* genes that present synonymous nucleotide diversity higher than 0.01 (all except *SFBB1*, *SFBB15*, and *SFBB18*; see Results) specific primers (Supplementary Table S6) were used to amplify these genes from genomic DNA of the 108 individuals analyzed from the three controlled crosses. For each *SFBB* gene, alleles present in the parents were used to select RFLPs that could be used to identify each of the *SFBB* alleles (Supplementary Table S8). It should be noted that, it is often not possible to develop a diagnostic marker for all four alleles segregating in a particular cross, since alleles of these genes have low levels of diversity.

### Phylogenetic analyses, summary statistics, recombination, and testing for positive selection at the *M. domestica SFBB* genes

*SFBB* sequences were deposited in GenBank (accession numbers MG458438-MG458668). These *SFBB* sequences together with those reported for *S3*-, and *S9*- and *S10*-haplotypes^23,27,44^, and the *SFBB* -lineage gene MDP0000250455 (not located in the *S*-locus, and not involved in GSI)^10^, used to root the phylogenetic tree, were aligned with Clustal Omega^82^. Maximum-likelihood trees were obtained with FastTree2^83^, using the general time reversible model with a proportion of invariant sites. A “CAT” rate for each site from among 20 fixed possibilities is first computed and then the lengths rescaled to optimize the gamma20 likelihood.

Analyses of DNA polymorphism, and minimum number of recombination events were performed using DnaSP v5^84^. The number of independent recombination events was inferred by RDP^85^ using the RDP, Chimaera, BootScan, 3Seq, GeneConv, MaxChi and SiScan methods (default options). A sequence is taken as recombinant if at least one of the methods identifies a recombination tract in that sequence with a probability smaller than 0.05. For each *SFBB* gene, the total number of synonymous mutations implied by the data was inferred using Yang’s^57^ methodology, under the appropriate model (M0 or M2; see Results), in ADOPS^86^.

For the identification of sites under positive selection we have used ADOPS^86^ and 10 datasets corresponding to *SFBB*s in each of the *S*-haplotypes here analyzed. Sequences were first aligned with the ClustalW2, and Muscle alignment algorithms as implemented in ADOPS^86^. Only codons with a support value above two are used for phylogenetic reconstruction. Bayesian trees were obtained using MrBayes, as implemented in the ADOPS pipeline^86^, using the GTR model of sequence evolution, allowing for among-site rate variation and a proportion of invariable sites. Third codon positions were allowed to have a gamma distribution shape parameter different from that of first and second codon positions. Two independent runs of 1,000,000 generations with four chains each (one cold and three heated chains) were set up. The average standard deviation of split frequencies was always about 0.01 and the potential scale reduction factor for every parameter about 1.00 showing that convergence has been achieved. Trees were sampled every 100th generation and the first 2500 samples were discarded (burn-in). The remaining trees were used to compute the Bayesian posterior probabilities of each clade of the consensus tree (see the B + database (bpositive.i3s.up.pt; see the Malus SFBB BP2017000011 dataset).We compare M2-M1 and M8-M7 models using codeML as implemented in ADOPS^86^. We consider as positively selected those amino acid sites that show a probability higher than 95% for both naive empirical Bayes (NEB) or Bayes empirical Bayes (BEB) methods in at least one of the analyses.

## Electronic supplementary material

Supplementary Material
