# Supplementary material for: Inferences on specificity recognition at the Malus×domestica gametophytic self-incompatibility system
Source: Sci Rep. 2018 Jan 29;8:1717. doi: 10.1038/s41598-018-19820-1 (PMC5788982; doi:10.1038/s41598-018-19820-1)
Supplement: Supplementary file 1 — Supplementary Material [file 41598_2018_19820_MOESM1_ESM.pdf]

Inferences on specificity recognition at the *Malus* × *domestica* gametophytic self-incompatibility system

Maria I. Pratas[1,2+], Bruno Aguiar[1,2+], Jorge Vieira[1,2+], Vanessa Nunes[1,2], Vanessa Teixeira[1,2], Nuno A. Fonseca[3], Amy Iezzoni[4], Steve van Nocker[4], and Cristina P. Vieira[1,2*]

Supplementary Information

Supplementary Table S1. Reads statistics.

Supplementary Table S2. FTKM of the *M. domestica* RefSeq *SFBB* genes

Supplementary Table S3. Trinity assembly statistics

Supplementary Table S4. Edena assembly statistics.

Supplementary Table S5. *SFBB* sequences obtained with primers SFBBgenF and SFBBgenR and genomic DNA of nine apple cultivars [‘Fuji’ (*S1*, *S9*), ‘Northern Spy’ (*S1*, *S3*), ‘Gala’ (*S2*, *S5*), ‘Golden Delicious’ (*S2*, *S3*), ‘Honeycrisp’ (*S2*, *S24*), ‘Idared’ (*S3*, *S7*), ‘Red Delicious’ (*S9*, *S28*), ‘McIntosh’ (*S10*, *S25*), and ‘Empire’ (*S10*, *S28*)] and those previously described for *S3-, S9*-, and *S10*-haplotypes [23,27,44].

Supplementary Table S6. Primers used to characterize the *SFBB* genes.

Supplementary Table S7. Summary of the segregation analyses of *M. domestica MdSFBB1/Sorbus SFBB13- MdSFBB12/SFBB10, SFBB14*, *SFBB16*, *SFBB17*, *SFBB19*, and *SFBB20* and their respective *S-RNase* in the progeny from three apple F1 populations [‘Golden Delicious’ (GD; *S2, S3*) × ‘Red Delicious’ (RD; *S9,S28*), ‘Gala’ (*S2, S5*) × ‘McIntosh’ (*S10, S25*), and ‘Fuji’ (*S1, S9*) × ‘Honeycrisp’ (*S2, S24*)].

Supplementary Table S8. Segregation analyses of *M. domestica MdSFBB1/Sorbus SFBB13-MdSFBB12/SFBB10, SFBB14*, *SFBB16*, *SFBB17*, *SFBB19*, and *SFBB20* in the progeny of

[‘Golden Delicious’ (GD; *S2, S3*) × ‘Red Delicious’ (RD; *S9,S28*), ‘Gala’ (*S2, S5*) × ‘McIntosh’ (*S10, S25*), and ‘Fuji’ (*S1, S9*) × ‘Honeycrisp’ (*S2, S24*)].

Supplementary Table S9. Primers used to characterize the *S-RNase* in the progeny from the crosses ‘McIntosh’ (*S10, S25*) × ‘Gala’ (*S2, S5*), ‘Fuji’ (*S1, S9*) × ‘Honeycrisp’ (*S2, S24*), and ‘Golden Delicious’ (*S2, S3*) × ‘Red Delicious’ (*S9, S28*).

Supplementary Fig. S1. Accumulation Curves for the anthers transcriptomes (A) and from other tissues (B).

Supplementary Fig. S2. Relative Expression (RTKM for a *SFBB* from a particular *S*-haplotype/ average RFKM values for the *SFBB* genes of that *S*-haplotype) of the 33 *SFBB* sequences previously identified for *S3*-, *S9*- and *S10*- haplotypes[23,27,44].

Supplementary Fig. S3. Relative Expression (RTKM for a *SFBB* from a particular S-haplotype/ average RFKM values for the *SFBB* genes of that *S*-haplotype) of the 22 *SFBB*s for *S1*-, *S2*-, *S3*-, *S9*-, *S10*-, and *S28*-haplotypes (those that are present in more than one cultivar analysed).

Supplementary Fig. S4. Amino acid sites identified as positively selected in the SFBB intra-haplotypic analyses (using two different alignment algorithms (C for ClustalW2 and M for Muscle) using the method of Yang[57] as implemented in ADOPS[86], with a probability higher than 95% in either NEB (naive empirical Bayes) or BEB (Bayes empirical Bayes)). The positively selected amino acid sites inferred for a given *S*-haplotype are shown on top of the SFBB4 reference sequence of the corresponding *S*-haplotype. yellow - NEB and BEB > 95%; red - NEB > 95% and BEB 90-95%; blue - NEB 90-95% and BEB > 95%. Stars indicate conserved amino acid sites and hashes the amino acid sites positively selected considered in this study. Different datasets were used when there are large differences in the length of the sequences. In brackets is shown the number of sequences analyzed.

Supplementary Fig. S5. Amino acid sites identified as positively selected in the Pyrinae (*Malus*, *Pyrus,* and *Sorbus*) S-RNases[61] (in red) on top of the alignment of the 10 S-RNases here analysed. In brackets are the GenBank accession numbers of these sequences used. Stars indicate conserved amino acid sites. C1 to C5 represent the conserved regions of the *S-RNase* gene.

Supplementary Table S1. Reads statistics

Tissue	Cultivar	Total number of reads	Read Length (bp)
Anther	Fuji (S1, S9)	10830000	101
	Northern Spy (NS; S1, S3)	5454784	101
	Golden Delicious (GD; S2, S3)	7027008	101
	Gala (S2, S5)	7599887	101
	Honeycrisp (Honey; S2, S24)	8303461	101
	Idared (S3, S7)	8190890	101
	Red Delicious (RD; S9, S28)	8735137	101
	McIntosh (MC; S10, S25)	8678121	101
	Empire (S10, S28)	9527733	101
Pollen	GD	6143121	101
Stigma	GD	6168860	101
Style	GD	6800753	101
Ovary	GD	7672506	101
Petals	GD	12430000	101
Sepals	GD	5091634	101
Filament	GD	1971828	101
Receptacle	GD	5769693	101
Young leaf	GD	7232869	101

Supplementary Table S2. FPKM of the S*FBB* genes annotated in the *M. domestica* genome

CDS	Homology (%)	Anthers					Pollen
		Gala (S2, S5)	GD (S2,S3)	Honey (S2, S24)	Idared (S3, S7)	Ns (S1, S3)	GD (S2,S3)
XP_008353030	100 (S*3-SFBB3* AB539850)	-	119.31	-	38.98	111.87	147.60
XP_008368690	95 (*S3-SFBB3,* AB539850)*	22.47	40.8	50.31	-	-	59.75
XP_008354461	99 (S*3-SFBB6;* AB539848)	-	50.79	-	49.49	31.78	57.35
XP_008338427	98 (*S3-SFBB6*)	-	3.58	-	-	-	9.69
XP_008351260	100 (S*3-SFBB7;* AB539845)	-	2.72	-	4.10	3.80	4.89
XP_008359869	97 (*S3-SFBB7;* AB539845)*	111.75	163.27	170.05	-	-	255.16
XP_008345368	100 (*S3-SFBB9;* AB539863)	-	52.55	-	1.32	0	33.38
XP_008386330	99 (*S3-MdSFBB11/SobusSFBB9;* AB539863)	-	89.14	-	125.38	79.60	77.95
XP_008362491	94 (*S3-MdSFBB9/SorbusSFBB11;* AB270795)*	26.96	36.60	42.38	-	-	57.43
XP_008353820	99 (*S3-MdSFBB1/SobusSFBB13;* AB539852)	-	46.48	-	50.10	58.00	46.25

* *SFBB* sequences showing homologies equal or below 97 % with *S3- SFBB* genes were assumed as *S2- SFBB* genes.

- not studied.

Supplementary Table S3. Trinity assembly statistics

Tissue	Cultivar	Number of contigs	N80	N50	N20	Size (bp) of the larger contig
Anther	Fuji (*S1*, *S9*)	97275	209	938	2023	9311
	NS (*S1*, *S3*)	76230	195	753	1795	6687
	GD (*S2*, *S3*)	81479	200	820	1875	8507
	Gala (*S2*, *S*5)	79593	221	939	1961	10998
	Honey (*S2*, *S24*)	77641	210	900	1976	9546
	Idared (*S3*, *S7*)	81785	228	976	1979	6841
	RD (*S9*, *S28*)	80919	211	905	1945	10314
	MC (*S10*, *S25*)	85778	219	970	2024	9281
	Empire (*S10*, *S28*)	85564	225	992	2040	9688
Pollen	GD	44976	190	723	1808	7083
Stigma	GD	83213	209	821	1742	6924
Style	GD	93991	211	869	1812	6001
Ovary	GD	99633	215	925	1882	8408
Petals	GD	207821	197	735	1946	9640
Sepals	GD	83444	210	822	1773	6381
Filament	GD	65845	160	385	1174	4973
Receptacle	GD	97675	197	761	1714	7442
Young leaf	GD	97305	227	945	1859	7928

Supplementary Table S4. Edena assembly statistics

Tissue	Cultivar	K-mer	Number of contigs	N80	N50	N20	Size (bp) of the larger contig
Anther	Fuji (*S1*, *S9*)	20	69856	123	181	349	2260
		60	51036	133	201	442	3286
	NS (*S1*, *S3*)	20	49039	126	189	348	1950
		60	27367	131	192	413	2965
	GD (*S2*, *S3*)	20	56284	124	186	350	1929
		60	35745	132	197	426	2956
	Gala (*S2*, *S5*)	20	60787	123	181	342	2024
		60	40564	132	199	424	2854
	Honey (*S2*, *S24*)	20	54606	124	185	352	1854
		60	37616	132	198	446	3011
	Idared (*S3*, *S7*)	20	63645	123	180	343	1969
		60	44147	132	198	423	3127
	RD (*S9*, *S28*)	20	58981	123	183	345	2344
		60	40547	133	200	444	3012
	MC (*S10*, *S25*)	20	62238	123	181	344	2477
		60	42992	132	199	437	2976
	Empire (*S10*, *S28*)	20	65336	123	180	342	2884
		60	46780	133	200	433	3090
Pollen	GD	20	26732	125	193	379	2136
		60	19481	135	206	480	3133
Stigma	GD	20	58905	126	191	357	2213
		60	31764	129	185	384	2677
Style	GD	20	67130	125	186	351	2388
		60	38417	130	191	398	2658
Ovary	GD	20	74264	124	182	346	2578
		60	45875	130	189	389	3130
Petals	GD	20	141999	138	268	556	3226
		60	65137	129	195	452	4241
Sepals	GD	20	57664	126	191	363	2233
		60	31053	130	191	399	3164
Filament	GD	20	26845	133	214	386	1601
		60	8991	126	179	357	1981
Receptacle	GD	20	62262	126	192	362	2235
		60	33648	129	184	368	3239
Young leaf	GD	20	77096	125	186	353	2017
		60	46271	129	183	362	2351

Supplementary Table S5. *SFBB* sequences obtained with primers SFBBgenF and SFBBgenR and genomicDNA of nine apple cultivars [‘Fuji’ (*S1*, *S9*), ‘Northern Spy’ (S1, *S3*), ‘Gala’ (*S2*, *S5*), ‘Golden Delicious’ (*S2*, *S3*), ‘Honeycrisp’ (*S2*, *S24*), ‘Idared’ (*S3*, *S7*), ‘Red Delicious’ (*S9*, *S28*), ‘McIntosh’ (*S10*, *S25*), and ‘Empire’ (*S10*, *S28*)] and those previously described for *S3-, S9-*, and *S10*-haplotypes (Sassa *et al.,* 2007; Minamikawa *et al.,* 2010; Okada *et al.,* 2013).

Gene	*M. domestica cultivars*								
	Fuji	Northern Spy	Gala	Golden Delicious	Honeycrisp	Idared	Red Delicious	McIntosh	Empire
MdSFBB1/SorbusSFBB13	*S1* (4)<br>*S9* (3; AB539859)	*S1* (2)<br>*S3* (6; AB539852)	*S5* (1)	*S3* (2; AB539852)	-	*S3* (1; AB539852)<br>*S7* (2)	*S9* (3; AB539859)	*S10* (3; AB699124)<br>*S25* (1)	*S28* (2)<br>AB699124 - *S10*
SFBB2	*S1* (2; HM013901*)<br>*S9* (1; AB699120)	*S3* (1; AB539857)	*S5* (2)	*S2*^{Z} (2)<br>*S3* (2; AB539857)	*S2*^{Z} (2)<br>*S24* (2)	*S3* (2; AB539857)<br>*S7* (2)	*S9* (2; AB699120)	*S25* (2; HM01391*)	*S28* (4)
SFBB3	-<br>AB539860 - S9	*S1* (1)<br>AB539850 - *S3*	*S2* (2)	*S2* (5)<br>AB539850 - *S3*	*S2* (4)<br>*S24* (4)	-<br>AB539850 - *S3*	**-**<br>AB539860 - *S9*	*S25* (1)<br>AB699123 - *S10*	-<br>AB699123 - *S10*
SFBB4	*S9* (3; AB699121)<br>DQ422810 *(*S1*)	*S1* (1; DQ422810*)<br>*S3* (4; AB539846)	*S5*(2)	*S3* (3; AB539846)	*S24* (3)	*S3* (1; AB539846)	*S9* (1; AB699121)<br>*S28* (7)	*S10* (2; AB699125)<br>*S25* (3)	*S10* (2; AB699125)<br>*S28* (4)

SFBB5	*S9* (2; AB270793)	*S1* (3)<br>AB539844(S3)	*S2* (2)<br>*S5* (4)	-<br>AB539844(S3)	*S2* (4)<br>*S24* (4)	AB539844(S3)<br>*S7* (5)	AB270793(S9)<br>*S28* (4)	*S25* (1)	*S10* (2)<br>*S28* (2)
SFBB6	*S1* (2; HM013899*)<br>*S9* (1; AB539858)	*S1* (5; HM013899*)<br>*S3* (4; AB539848)	*S5* (3; AB539848)	*S3* (5; AB539848)	*S2* (5)<br>*S24* (1)	*S7* (4)	*S28* (3)<br>AB539858- *S9*	*S10* (7; AB699128)<br>*S25* (2; HM013914*)	*S10* (2; AB699128)
SFBB7	*S1* (1)<br>AB270794 - *S9*	*S1* (6)<br>*S3* (2; AB539845)	-	*S3* (1; AB539845)	*S24* (3)	*S7* (1)<br>AB539845 - *S3*	-<br>AB270794 - *S9*	*S10-2* (1)<br>*S10-1* (2- HM013924*)	*S10-1* (1; HM013924*)
SFBB8	*S1* (1; HM013904*)<br>AB539853 - *S9*	*S1* (2; HM013904*)<br>AB539861- *S3*	*S5* (1)	-<br>AB539861 - *S3*	*S24* (2)	*S7* (1)<br>AB539861 - *S3*	*S28* (1)<br>AB539853 - *S9*	-<br>AB699129 - *S10*	*S28* (2)<br>AB699129 - *S10*
MdSFBB9/SorbusSFBB11	-<br>AB539862 - *S9*	*S1* (2)<br>AB270795 - *S3*	*S2* (2)	*S2* (3)<br>*S3* (1; AB270795)	*S2* (4)<br>*S24* (5)	*S3* (1; AB270795)<br>*S7* (6)	-<br>AB539862 - *S9*	*S10* (6; AB699126)<br>*S25* (1)	*S10* (3; AB699126)
MdSFBB10/SorbusSFBB12	*S1* (2)<br>AB539849 - *S9*	*S1* (1)<br>AB270796 - *S3*	-	-<br>AB270796 - *S3*	*S2* (3)<br>*S24* (3)	-<br>AB270796 - *S3*	*S28* (2)<br>AB539849 - *S9*	*S25* (2; HM013922*)	*S28* (2)

MdSFBB11/SorbusSFBB9	*S1* (5)<br>*S9* (5; AB539855)	*S1* (3)<br>*S3* (4; AB539863)	*S2* (1)	*S3* (3; AB539863)	*S24* (1)	*S3* (2; AB539863)	*S9* (3; AB539855)<br>*S28* (1)	*S10* (6)<br>*S25* (3)	*S10* (4)<br>*S28* (2)
MdSFBB12/SorbusSFBB10	*S1* (2)<br>AB539856 - *S9*	-	-	*S2* (1)	-	-	*S28* (1)<br>AB539856 - *S9*	*S25* (4)	*S28* (4)
MdSFBB13/SobusSFBB1	*S1* (3)	*S1* (4)<br>*S3* (2)	*S5* (3)	*S2* (1)<br>*S3* (1)	*S2* (4)	*S3* (1)<br>*S7* (3)	*S28* (1)	*S10* (1)	*S10* (1)<br>*S28* (1)
SFBB14	*S1* (3)<br>*S9* (3)	*S1* (4)<br>*S3* (1)	*S2* (2)	*S2* (1)<br>*S3* (4)	*S2* (4)<br>*S24* (3)	*S3* (1)	*S9* (1)	*S25* (3)	-
SFBB15	T1 (4)<br>T2(1)	T1 (4)	T1 (1)	T1 (2)	T3 (3)	T1 (3)<br>T3 (3)	T1 (1)<br>T3 (1)	T4 (1)<br>T5 (2)	T2 (6)
SFBB16	-	*S1* (1)<br>AB539851 - *S3*	*S2* (2)<br>*S5* (1)	*S2* (2)<br>AB539851 - *S3*	*S2* (2)<br>*S24* (3)	*S7* (1)<br>AB539851 - *S3*	*S28* (2)	*S25* (1)	*S28* (2)
SFBB17	*S1* (2)	-	*S5* (1)	*S2* (1)	*S24* (1)	*S7* (3)<br>AB539854 - *S3*; -	*S28* (2)<br>AB539854 - *S3*; -	*S10* (1)	*S28* (3)

	AB539854 - *S3*; -*S9*	AB539854 -*S3*; -*S9*		AB539854 - *S3*; -*S9*		*S9*	*S9*	*S25* (1)	
	S1 (2)			*S2* (1)			*S28*(1)		
SFBB18	*S9* (1; HM013903*)	*S1* (2)		*S3* (1)	*S24* (1)	*S3* (1)	HM013903* - *S9*		*S10* (1)
			-					-	
SFBB19	*S9* (2; AB699122)								
							S9 (1; AB699122)		
SFBB20							*S28*(2)		*S28*(3)
									S10 (1)
Pseudogenes			*S2* (1)		*S2*(1)		*S28* (3)	*S10*(3)	S28 (2)

Underline sequences are those reported for *S3*-, *S9*-, and *S1*0-haplotypes not obtained using primers SFBgenF and SFBBgenR. Stars indicate GenBank *SFBB* sequences obtained from other cultivars that present a common *S*-haplotype with cultivars here used. The Z represents *S2*-*SFBB2* sequence that has a nucleotide insertion that creates in frame stop codons. Double underline represent GenBank *SFBB* sequences obtained from, that present a common *S*-haplotype with cultivars here used, not obtained in this study. In brackets are the number of colonies sequenced that present a particular sequence

Supplementary Table S6. Primers used to characterized the *SFBB* genes

Name	Primer		Amplification	Annealing
	Forward	Reverse	product (bp)	temperature (ºC)
SFBBgen*	AAGTCYCTGATGMGRTTC	GTCCATTACCCAYRTYTC	870 to 889	48
MdSFBB13/SorbusSFBB1*#	CTCTTATCAATAGTCCAT	AAGGTCCAGCAGTTAGGT	897	48
SFBB2#	SFBBgenF	CATTACCCATATTTCAAG	882	49
SFBB2-SE*+	CCCTCATCAATAGTCCTC	AGAAAAATACCATCAAGC	767	49
SFBB3#	CATCAATAGTCCAAGTTT	SFBBgenR	822	49
SFBB3-SE+	TTCTCAACCGTTCTCAGT	AATCAAATGAAAGTATGC	588	52
SFBB4#	GCAACTTCCTGATTCATT	TTCCTCACCATCCCTTGT	324	49
SFBB4-SE+	GCAACTTCCTGATTCATT	TTCTTCACCATCCCTTGT	325	49
	FujixHoney and GalaxMcIntosh			
	SFBB4F	SFBBgenR	484	52
SFBB5-SE+	GTGAAACTCCTGAAGATC	CCTAGTGTCAATGGATAA	1015	49
SFBB6-SE+	TTCTCATCCTTCACTTGC	AACCTCGTCACATTTCCA	879	50
SFBB6-SE FujixHoney +	ACTTCACTCGCATCCTTT	AAATCCCTTCAAGTACAT	535	49
SFBB7#	ACAACAAACTCTCATCCR	CGATAGCAATACGAAGTS	741	49
		AAGCAATAYGAAGTGAC		
SFBB7-SE+	CATCAATAGTCCAAGTTT	A	784	50
SFBB8#	CTCTTATCAATAGTCCAT	SFBBgenR	845	49
SFBB8-SE+	CTCTTATCAATAGTCCAT	AAGGTCCAGCAGTTAGGT	897	49
>MdSFBB11/SorbusSFBB9#	GTATCCTTCTCAACCGTA	GCCAATAACAAAATCCCT	561	48
MdSFBB11/SorbusSFBB9-SE+	TGTATCCTTCTCAACCGT	CTCGCCATCGTTTGCAAG	579	52
	S5; S24	S5; S24		
MdSFBB12/SorbusSFBB10#	GTTCAGATTCACGGTTAC	GTAAGGGATTCATTACGA	527	49
		S7		
	S7 CCTCCACTTGTATCCTTG	ATCCATTACCCATATTTC	764	49
MdSFBB12/SorbusSFBB10+	ACTCTCATCCTCyACTTG	GTAAGGGATTCATTACGA	708	49
MdSFBB9/SorbusSFBB11#	SFBBgenF	ATAACAAAATCCCTTCAT	672	50
MdSFBB9/SorbusSFBB11-SE+	SFBBgenF	ATAACAAAATCCCTTCAT	672	54
MdSFBB10/SorbusSFBB12#	CGTTCTCAGGCTCACATT	S5; S10 SFBBgenR	737	49
		S7		
		GCAAGGGATTCATTTCGG	674	50

MdSFBB10/SorbusSFBB12-SE+	CGTTCTCAGGCTCACATT	GCAAGGGATTCATTTCGG	674	51
MdSFBB1/SorbusSFBB13#	AATTCAGGCAACTTCCSC	AAGTATGTATTTCTCGCC	341	49
MdSFBB1/SorbusSFBB13-SE+	ATTCAGGCAACTTCCCCC	TTAGAAAACTCAAAATCC	417	52
SFBB14#	AACCGTTCTCAGATGCCGG	CCTCAGTTGGATCATAAT	712	50
SFBB14-SE+	CAGGGAAAACTGTTATTA	AAGAAGTGATGGATTCAT	489	52
SFBB16-SE+	CCCTCATCAATAGTCCTC	TGAGGAAGAGCAGTATGT	525	49
SFBB17-SE+	SFBBgenF	TTTACTCCATCATAGTTG	890	49
SFBB20	GACAACAAACTCTCATCG	TCCGTCATAGTCATCCAA	810	49
	GACAACAAACTCTCATCG	SFBBgenR	798	49
	SFBBgenF	TCCGTCATAGTCATCCAA	898	50
SFBB24	CTCATCCTCCACTTGTAT	GGTTTCTCAATGCCTTTC	852	49

* Primers described in [22]

# used to characterize *S*-haplotypes not amplified with SFBBgen primers

+ used to characterize the progeny from the crosses ‘McIntosh’ (*S10, S25*) × ‘Gala’ (*S2, S5*), ‘Fuji’ (*S1, S9*) × ‘Honeycrisp’ (*S2, S24*), and ‘Golden Delicious’ (*S2, S3*) × ‘Red Delicious’ (*S9, S28*).

Supplementary Table S7. Summary of the segregation analyses of *M. domestica MdSFBB1/Sorbus SFBB13- MdSFBB12/SFBB10, SFBB14*, *SFBB16*, *SFBB17*, *SFBB19*, and *SFBB20* and their respective *S-RNase* in the progeny from three apple F1 populations [‘Golden Delicious’ (GD; *S2, S3*) × ‘Red Delicious’ (RD; *S9,S28*), ‘Gala’ (*S2, S5*) × ‘McIntosh’ (*S10, S25*), and ‘Fuji’ (*S1, S9*) × ‘Honeycrisp’ (*S2, S24*)].

Gene	Allele	Cross	number of recombinant gametes
MdSFBB1/SorbusSFBB13	*S9*	GD × RD	0 /16
	S28	GD × RD	0 /11
	S1	Fuji × Honeycrisp	0 /17
SFBB2	*S3*	GD × RD	0/14
	S9	GD × RD	0 /16
	S5	Gala × McIntosh	0 /19
	S25	Gala × McIntosh	0 /22
SFBB3	*S28*	GD × RD	0 /11
	S2	Gala × McIntosh	0 /29
	S10	Gala × McIntosh	0 /26
	S2	Fuji × Honeycrisp	0 /20
SFBB4	*S28*	GD × RD	0 /11
	S5	Gala × McIntosh	0 /19
	S10	Gala × McIntosh	0 /26
	S25	Gala × McIntosh	0 /22
	S1	Fuji × Honeycrisp	0 /17
SFBB5	*S28*	GD × RD	0 /11
	S5	Gala × McIntosh	0 /19
	S10	Gala × McIntosh	0 /26
SFBB6	*S3*	GD × RD	0 /14

	S9	GD × RD	0 /16
	S28	GD × RD	0 /11
	S1	Fuji × Honeycrisp	0 /17
	S25	Gala × McIntosh	0 /22
SFBB7	*S9*	Fuji × Honeycrisp	0 /17
	S24	Fuji × Honeycrisp	0 /14
SFBB8	*S1*	Fuji × Honeycrisp	0 /17
	S2	Fuji × Honeycrisp	0 /20
	S9	Fuji × Honeycrisp	0 /17
	S24	Fuji × Honeycrisp	0 /14
	S2	Gala × McIntosh	0 /29
	S5	Gala × McIntosh	0 /19
	S10	Gala × McIntosh	0 /26
MdSFBB9/SorbusSFBB11	*S1*	Fuji × Honeycrisp	0 /17
	S9	Fuji × Honeycrisp	0 /17
	S24	Fuji × Honeycrisp	0 /14
	S5	Gala × McIntosh	0 /19
	S10	Gala × McIntosh	0 /26
MdSFBB10/SorbusSFBB12	*S9*	GD × RD	0 /16
	S28	GD × RD	0 /11
	S1	Fuji × Honeycrisp	0 /17
	S2	Fuji × Honeycrisp	0 /20
	S9	Fuji × Honeycrisp	0 /17
	S24	Fuji × Honeycrisp	0 /14
MdSFBB11SorbusSFBB9	*S2*	GD × RD	0 /13
	S9	GD × RD	0 /16
	S28	GD × RD	0 /11

	S1	Fuji × Honeycrisp	0 /17
	S2	Fuji × Honeycrisp	0 /20
	S9	Fuji × Honeycrisp	0 /17
	S24	Fuji × Honeycrisp	0 /14
	S10	Gala × McIntosh	0 /26
MdSFBB12/SorbusSFBB10	*S1*	Fuji × Honeycrisp	0 /17
	S2	Fuji × Honeycrisp	0 /20
	S9	Fuji × Honeycrisp	0 /17
SFBB14	S9	Fuji × Honeycrisp	0 /17
	S10	Gala × McIntosh	0 /26
	S25	Gala × McIntosh	0 /22
SFBB16	*S3*	GD × RD	0 /14
	S1	Fuji × Honeycrisp	0 /17
	S2	Fuji × Honeycrisp	0 /20
	S24	Fuji × Honeycrisp	0 /14
	S10	Gala × McIntosh	0 /26
	S25	Gala × McIntosh	0 /22
SFBB17	*S10*	Gala × McIntosh	0 /26
	S25	Gala × McIntosh	0 /22
SFBB19	*S9*	GD × RD	0 /16
SFBB20	*S28*	GD × RD	0 /11

Supplementary Table S8. Segregation analyses of *M. domestica* *MdSFBB1/SorbusSFBB13-MdSFBB12/SorbusSFBB10*, *SFBB14*, *SFBB16*, *SFBB17*, *SFBB19*, and *SFBB20* in the progeny of Golden Delicious (GD; *S2*, *S3*) x Red Delicious (RD; *S9*, *S28*), Gala (*S2*, *S5*) x McIntosh (*S10*, *S25*), and Fuji (*S1*, *S9*) x Honeycrisp (*S2*, *S24*), based on the RFLP for each *SFBB* allele.

Individual	S- haplotpe		MdSFBB1/SobusSFBB13			SFBB2				SFBB3			SFBB4					SFBB5		
			S1-	S9-	S28-	S3-	S5-	S9-	S25-	S2-	S10-	S28-	S1-	S5-	S10-	S25-	S28-	S5-	S10-	S28-
			RsaI	MaeIII	RsaI	MaeII	ApoI	MaeII	BclI	DdeI	TatI	MnlI	MseI	DdeI	MseI	DdeI	EciI	BccI	BsmI	HphI
			138 150	74 307	381	236 646	221 243 418	184 698	279 603	270 305	495	194	159 339	280	214 284	157 303	325	252 624	641	273 315
GDXRD 4	S3	S28		...	x	x		...				x					x			x
GDXRD 5	S3	S28		...	x	x		...				x					x			x
GDXRD 6	S3	S28		...	x	x		...				x					x			x
GDXRD 7	S2	S9		x	...	...		x				...					...			...
GDXRD 8	S2	S28		...	x	...		...				x					x			x
GDXRD 9	S2	S9		x	...	...		x				...					...			...
GDXRD 11	S3	S9		x	...	x		x				...					...			...
GDXRD 12	S2	S28		...	x	...		...				x					x			x
GDXRD 13	S3	S28		...	x	x		...				x					x			x
GDXRD 15	S3	S9		x	...	x		x				...					...			...
GDXRD 16	S2	S28		...	x	...		...				x					x			x
GDXRD 18	S2	S28		...	x	...		...				x					x			x
GDXRD 20	S3	S9		x	...	x		x				...					...			...
GDXRD 22	S2	S9		x	...	...		x				...					...			...
GDXRD 24	S2	S9		x	...	...		x				...					...			...
GDXRD 25	S2	S9		x	...	...		x				...					...			...
GDXRD 26	S2	S28		...	x	...		...				x					x			x
GDXRD 28	S3	S9		x	...	x		x				...					...			...
GDXRD 29	S3	S9		x	...	x		x				...					...			...
GDXRD 30	S2	S28		...	x	...		...				x					x			x
GDXRD 31	S3	S9		x	...	x		x				...					...			...
GDXRD 32	S3	S9		x	...	x		x				...					...			...
GDXRD 33	S2	S9		x	...	...		x				...					...			...
GDXRD 34	S3	S28		...	x	x		...				x					x			x
GDXRD 35	S3	S9		x	...	x		x				...					...			...
GDXRD 36	S2	S9		x	...	...		x				...					...			...
GDXRD 37	S3	S9		x	...	x		x				...					...			...
GalaXMc 5	S5	S10					x		...	...	x			x	x	...		x	x	
GalaXMc 6	S2	S10					...		...	x	x			...	x	...		...	x	
GalaXMc 7	S2	S25					...		x	x	...			...	...	x		...	...	
GalaXMc 8	S2	S25					...		x	x	...			...	...	x		...	...	
GalaXMc 9	S5	S25					x		x	...	...			x	...	x		x	...	
GalaXMc 10	S5	S10					x		...	...	x			x	x	...		x	x	
GalaXMc 11	S2	S10					...		...	x	x			...	x	...		...	x	
GalaXMc 13	S2	S25					...		x	x	...			...	...	x		...	...	
GalaXMc 14	S5	S10					x		...	...	x			x	x	...		x	x	
GalaXMc 16	S2	S25					...		x	x	...			...	...	x		...	...	
GalaXMc 17	S2	S10					...		...	x	x			...	x	...		...	x	
GalaXMc 18	S2	S25					...		x	x	...			...	...	x		...	...	
GalaXMc 21	S5	S10					x		...	...	x			x	x	...		x	x	
GalaXMc 28	S5	S10					x		...	...	x			x	x	...		x	x	
GalaXMc 29	S2	S10					...		...	x	x			...	x	...		...	x	
GalaXMc 30	S2	S10					...		...	x	x			...	x	...		...	x	
GalaXMc 31	S2	S10					...		...	x	x			...	x	...		...	x	
GalaXMc 36	S5	S25					x		x	...	...			x	...	x		x	...	
GalaXMc 38	S2	S25					...		x	x	...			...	...	x		...	...	
GalaXMc 39	S2	S25					...		x	x	...			...	...	x		...	...	
GalaXMc 40	S5	S10					x		...	...	x			x	x	...		x	x	
GalaXMc 41	S2	S25					...		x	x	...			...	...	x		...	...	
GalaXMc 42	S5	S10					x		...	...	x			x	x	...		x	x	
GalaXMc 44	S2	S25					...		x	x	...			...	...	x		...	...	
GalaXMc 45	S2	S25					...		x	x	...			...	...	x		...	...	
GalaXMc 46	S2	S10					...		...	x	x			...	x	...		...	x	
GalaXMc 47	S5	S10					x		...	...	x			x	x	...		x	x	
GalaXMc 48	S5	S10					x		...	...	x			x	x	...		x	x	
GalaXMc 50	S5	S10					x		...	...	x			x	x	...		x	x	
GalaXMc 51	S5	S25					x		x	...	...			x	...	x		x	...	
GalaXMc 52	S2	S10					...		...	x	x			...	x	...		...	x	
GalaXMc 53	S2	S10					...		...	x	x			...	x	...		...	x	
GalaXMc 54	S5	S10					x		...	...	x			x	x	...		x	x	
GalaXMc 55	S2	S25					...		x	x	...			...	...	x		...	...	
GalaXMc 56	S2	S10					...		...	x	x			...	x	...		...	x	
GalaXMc 57	S2	S10					...		...	x	x			...	x	...		...	x	
GalaXMc 58	S5	S25					x		x	...	...			x	...	x		x	...	
GalaXMc 60	S2	S10					...		...	x	x			...	x	...		...	x	
GalaXMc 63	S5	S25					x		x	...	...			x	...	x		x	...	
GalaXMc 65	S5	S10					x		...	...	x			x	x	...		x	x	
GalaXMc 66	S2	S25					...		x	x	...			...	...	x		...	...	
GalaXMc 67	S2	S25					...		x	x	...			...	...	x		...	...	
GalaXMc 69	S2	S10					...		...	x	x			...	x	...		...	x	
GalaXMc 71	S5	S25					x		x	...	...			x	...	x		x	...	
GalaXMc 72	S2	S25					...		x	x	...			...	...	x		...	...	
GalaXMc 74	S5	S25					x		x	...	...			x	...	x		x	...	
GalaXMc 75	S2	S10					...		...	x	x			...	x	...		...	x	
GalaXMc 76	S2	S25					...		x	x	...			...	...	x		...	...	
FujiXHoney 2	S1	S2	x							x			x							
FujiXHoney 3	S2	S9	...							x			...							
FujiXHoney 5	S2	S9	...							x			...							
FujiXHoney 6	S1	S24	x							...			x							
FujiXHoney 7	S2	S9	...							x			...							
FujiXHoney 8	S9	S24	...							...			...							
FujiXHoney 9	S1	S2	x							x			x							
FujiXHoney 10	S1	S24	x							...			x							
FujiXHoney 12	S1	S24	x							...			x							
FujiXHoney 13	S2	S9	...							x			...							
FujiXHoney 14	S2	S9	...							x			...							
FujiXHoney 15	S1	S24	x							...			x							
FujiXHoney 16	S2	S9	...							x			...							
FujiXHoney 18	S2	S9	...							x			...							
FujiXHoney 19	S2	S9	...							x			...							
FujiXHoney 20	S1	S24	x							...			x							
FujiXHoney 23	S1	S2	x							x			x							
FujiXHoney 26	S9	S24	...							...			...							
FujiXHoney 27	S2	S9	...							x			...							
FujiXHoney 31	S2	S9	...							x			...							
FujiXHoney 32	S1	S24	x							...			x							
FujiXHoney 33	S1	S2	x							x			x							
FujiXHoney 35	S1	S24	x							...			x							
FujiXHoney 36	S1	S2	x							x			x							
FujiXHoney 37	S9	S24	...							...			...							
FujiXHoney 40	S2	S9	...							x			...							
FujiXHoney 45	S1	S2	x							x			x							
FujiXHoney 48	S2	S9	...							x			...							
FujiXHoney 49	S1	S2	x							x			x							
FujiXHoney 50	S9	S24	...							...			...							
FujiXHoney 51	S1	S24	x							...			x							
FujiXHoney 52	S1	S24	x							...			x							
FujiXHoney 53	S1	S2	x							x			x							
FujiXHoney 54	S9	S24	...							...			...							

Individual	S- haplotpe		SFBB6					SFBB7		SFBB8						MdSFBB9/SobusSFBB11				
			S1-	S3-	S9-	S25-	S28-	S9-	S24-	S1-	S2-	S5-	S9-	S10-	S24-	S1-	S5-	S9-	S10-	S24-
			NcoI	BsrDI	HphI	RsaI	TatI	HphI	HpaII	DdeI	Eco57I	AccI	HpaII	MaeIII	ApoI	ApoI	Sau3AI	MmeI	MwoI	ApoI
			280 300	879	330 336	233	230	330 368	792	122	365 433	344 535	250	104 168	300	418	402	179 366	672	300
GDXRD 4	S3	S28		x	...		x													
GDXRD 5	S3	S28		x	...		x													
GDXRD 6	S3	S28		x	...		x													
GDXRD 7	S2	S9		...	x		...													
GDXRD 8	S2	S28		...	...		x													
GDXRD 9	S2	S9		...	x		...													
GDXRD 11	S3	S9		x	x		...													
GDXRD 12	S2	S28		...	...		x													
GDXRD 13	S3	S28		x	...		x													
GDXRD 15	S3	S9		x	x		...													
GDXRD 16	S2	S28		...	...		x													
GDXRD 18	S2	S28		...	...		x													
GDXRD 20	S3	S9		x	x		...													
GDXRD 22	S2	S9		...	x		...													
GDXRD 24	S2	S9		...	x		...													
GDXRD 25	S2	S9		...	x		...													
GDXRD 26	S2	S28		...	...		x													
GDXRD 28	S3	S9		x	x		...													
GDXRD 29	S3	S9		x	x		...													
GDXRD 30	S2	S28		...	...		x													
GDXRD 31	S3	S9		x	x		...													
GDXRD 32	S3	S9		x	x		...													
GDXRD 33	S2	S9		...	x		...													
GDXRD 34	S3	S28		x	...		x													
GDXRD 35	S3	S9		x	x		...													
GDXRD 36	S2	S9		...	x		...													
GDXRD 37	S3	S9		x	x		...													
GalaXMc 5	S5	S10				...					...	x		x			x		x	
GalaXMc 6	S2	S10				...					x	...		x			...		x	
GalaXMc 7	S2	S25				x					x	...		...			...		...	
GalaXMc 8	S2	S25				x					x	...		...			...		...	
GalaXMc 9	S5	S25				x					...	x		...			x		...	
GalaXMc 10	S5	S10				...					...	x		x			x		x	
GalaXMc 11	S2	S10				...					x	...		x			...		x	
GalaXMc 13	S2	S25				x					x	...		...			...		...	
GalaXMc 14	S5	S10				...					...	x		x			x		x	
GalaXMc 16	S2	S25				x					x	...		...			...		...	
GalaXMc 17	S2	S10				...					x	...		x			...		x	
GalaXMc 18	S2	S25				x					x	...		...			...		...	
GalaXMc 21	S5	S10				...					...	x		x			x		x	
GalaXMc 28	S5	S10				...					...	x		x			x		x	
GalaXMc 29	S2	S10				...					x	...		x			...		x	
GalaXMc 30	S2	S10				...					x	...		x			...		x	
GalaXMc 31	S2	S10				...					x	...		x			...		x	
GalaXMc 36	S5	S25				x					...	x		...			x		...	
GalaXMc 38	S2	S25				x					x	...		...			...		...	
GalaXMc 39	S2	S25				x					x	...		...			...		...	
GalaXMc 40	S5	S10				...					...	x		x			x		x	
GalaXMc 41	S2	S25				x					x	...		...			...		...	
GalaXMc 42	S5	S10				...					...	x		x			x		x	
GalaXMc 44	S2	S25				x					x	...		...			...		...	
GalaXMc 45	S2	S25				x					x	...		...			...		...	
GalaXMc 46	S2	S10				...					x	...		x			...		x	
GalaXMc 47	S5	S10				...					...	x		x			x		x	
GalaXMc 48	S5	S10				...					...	x		x			x		x	
GalaXMc 50	S5	S10				...					...	x		x			x		x	
GalaXMc 51	S5	S25				x					...	x		...			x		...	
GalaXMc 52	S2	S10				...					x	...		x			...		x	
GalaXMc 53	S2	S10				...					x	...		x			...		x	
GalaXMc 54	S5	S10				...					...	x		x			x		x	
GalaXMc 55	S2	S25				x					x	...		...			...		...	
GalaXMc 56	S2	S10				...					x	...		x			...		x	
GalaXMc 57	S2	S10				...					x	...		x			...		x	
GalaXMc 58	S5	S25				x					...	x		...			x		...	
GalaXMc 60	S2	S10				...					x	...		x			...		x	
GalaXMc 63	S5	S25				x					...	x		...			x		...	
GalaXMc 65	S5	S10				...					...	x		x			x		x	
GalaXMc 66	S2	S25				x					x	...		...			...		...	
GalaXMc 67	S2	S25				x					x	...		...			...		...	
GalaXMc 69	S2	S10				...					x	...		x			...		x	
GalaXMc 71	S5	S25				x					...	x		...			x		...	
GalaXMc 72	S2	S25				x					x	...		...			...		...	
GalaXMc 74	S5	S25				x					...	x		...			x		...	
GalaXMc 75	S2	S10				...					x	...		x			...		x	
GalaXMc 76	S2	S25				x					x	...		...			...		...	
FujiXHoney 2	S1	S2	x					...	...	x	x		...		...	x		...		...
FujiXHoney 3	S2	S9	...					x	...	...	x		x		...	...		x		...
FujiXHoney 5	S2	S9	...					x	...	...	x		x		...	...		x		...
FujiXHoney 6	S1	S24	x					...	x	x	...		...		x	x		...		x
FujiXHoney 7	S2	S9	...					x	...	...	x		x		...	...		x		...
FujiXHoney 8	S9	S24	...					x	x	...	...		x		x	...		x		x
FujiXHoney 9	S1	S2	x					...	...	x	x		...		...	x		...		...
FujiXHoney 10	S1	S24	x					...	x	x	...		...		x	x		...		x
FujiXHoney 12	S1	S24	x					...	x	x	...		...		x	x		...		x
FujiXHoney 13	S2	S9	...					x	...	...	x		x		...	...		x		...
FujiXHoney 14	S2	S9	...					x	...	...	x		x		...	...		x		...
FujiXHoney 15	S1	S24	x					...	x	x	...		...		x	x		...		x
FujiXHoney 16	S2	S9	...					x	...	...	x		x		...	...		x		...
FujiXHoney 18	S2	S9	...					x	...	...	x		x		...	...		x		...
FujiXHoney 19	S2	S9	...					x	...	...	x		x		...	...		x		...
FujiXHoney 20	S1	S24	x					...	x	x	...		...		x	x		...		x
FujiXHoney 23	S1	S2	x					...	...	x	x		...		...	x		...		...
FujiXHoney 26	S9	S24	...					x	x	...	...		x		x	...		x		x
FujiXHoney 27	S2	S9	...					x	...	...	x		x		...	...		x		...
FujiXHoney 31	S2	S9	...					x	...	...	x		x		...	...		x		...
FujiXHoney 32	S1	S24	x					...	x	x	...		...		x	x		...		x
FujiXHoney 33	S1	S2	x					...	...	x	x		...		...	x		...		...
FujiXHoney 35	S1	S24	x					...	x	x	...		...		x	x		...		x
FujiXHoney 36	S1	S2	x					...	...	x	x		...		...	x		...		...
FujiXHoney 37	S9	S24	...					x	x	...	...		x		x	...		x		x
FujiXHoney 40	S2	S9	...					x	...	...	x		x		...	...		x		...
FujiXHoney 45	S1	S2	x					...	...	x	x		...		...	x		...		...
FujiXHoney 48	S2	S9	...					x	...	...	x		x		...	...		x		...
FujiXHoney 49	S1	S2	x					...	...	x	x		...		...	x		...		...
FujiXHoney 50	S9	S24	...					x	x	...	...		x		x	...		x		x
FujiXHoney 51	S1	S24	x					...	x	x	...		...		x	x		...		x
FujiXHoney 52	S1	S24	x					...	x	x	...		...		x	x		...		x
FujiXHoney 53	S1	S2	x					...	...	x	x		...		...	x		...		...
FujiXHoney 54	S9	S24	...					x	x	...	...		x		x	...		x		x

Individual	S- haplotpe		MdSFBB10/SobusSFBB12					MdSFBB11/SobusSFBB9								MdSFBB12/SobusSFBB10		
			S1-	S2-	S9-	S24-	S28-	S1-	S2-		S9-		S10-	S24-	S28-	S1-	S2-	S9-
			TaqI	MmeI	HinfI	NlaIII	HinfI	MaeII	HpaII*	Sdu#	RsaI*	MboI#	GsuI	AvaII	RsaI	MaeII	NcoI	TaqI
			350	253 421	98 527	190	109	217 351	132 163 284	357	256 295	126 194	278 300	108	119 442	129	252 456	78
GDXRD 4	S3	S28			...		x		...		...				x			
GDXRD 5	S3	S28			...		x		...		...				x			
GDXRD 6	S3	S28			...		x		...		...				x			
GDXRD 7	S2	S9			x		...		x		x				...			
GDXRD 8	S2	S28			..		x		x		...				x			
GDXRD 9	S2	S9			x		...		x		x				...			
GDXRD 11	S3	S9			x		...		...		x				...			
GDXRD 12	S2	S28			...		x		x		...				x			
GDXRD 13	S3	S28			...		x		...		...				x			
GDXRD 15	S3	S9			x		...		...		x				...			
GDXRD 16	S2	S28			...		x		x		...				x			
GDXRD 18	S2	S28			...		x		x		...				x			
GDXRD 20	S3	S9			x		...		...		x				...			
GDXRD 22	S2	S9			x		...		x		x				...			
GDXRD 24	S2	S9			x		...		x		x				...			
GDXRD 25	S2	S9			x		...		x		x				...			
GDXRD 26	S2	S28			...		x		x		...				x			
GDXRD 28	S3	S9			x		...		...		x				...			
GDXRD 29	S3	S9			x		...		...		x				...			
GDXRD 30	S2	S28			...		x		x		...				x			
GDXRD 31	S3	S9			x		...		...		x				...			
GDXRD 32	S3	S9			x		...		...		x				...			
GDXRD 33	S2	S9			x		...		x		x				...			
GDXRD 34	S3	S28			...		x		...		...				x			
GDXRD 35	S3	S9			x		...		...		x				...			
GDXRD 36	S2	S9			x		...		x		x				...			
GDXRD 37	S3	S9			x		...		...		x				...			
GalaXMc 5	S5	S10											x					
GalaXMc 6	S2	S10											x					
GalaXMc 7	S2	S25											...					
GalaXMc 8	S2	S25											...					
GalaXMc 9	S5	S25											...					
GalaXMc 10	S5	S10											x					
GalaXMc 11	S2	S10											x					
GalaXMc 13	S2	S25											...					
GalaXMc 14	S5	S10											x					
GalaXMc 16	S2	S25											...					
GalaXMc 17	S2	S10											x					
GalaXMc 18	S2	S25											...					
GalaXMc 21	S5	S10											x					
GalaXMc 28	S5	S10											x					
GalaXMc 29	S2	S10											x					
GalaXMc 30	S2	S10											x					
GalaXMc 31	S2	S10											x					
GalaXMc 36	S5	S25											...					
GalaXMc 38	S2	S25											...					
GalaXMc 39	S2	S25											...					
GalaXMc 40	S5	S10											x					
GalaXMc 41	S2	S25											...					
GalaXMc 42	S5	S10											x					
GalaXMc 44	S2	S25											...					
GalaXMc 45	S2	S25											...					
GalaXMc 46	S2	S10											x					
GalaXMc 47	S5	S10											x					
GalaXMc 48	S5	S10											x					
GalaXMc 50	S5	S10											x					
GalaXMc 51	S5	S25											...					
GalaXMc 52	S2	S10											x					
GalaXMc 53	S2	S10											x					
GalaXMc 54	S5	S10											x					
GalaXMc 55	S2	S25											...					
GalaXMc 56	S2	S10											x					
GalaXMc 57	S2	S10											x					
GalaXMc 58	S5	S25											...					
GalaXMc 60	S2	S10											x					
GalaXMc 63	S5	S25											...					
GalaXMc 65	S5	S10											x					
GalaXMc 66	S2	S25											...					
GalaXMc 67	S2	S25											...					
GalaXMc 69	S2	S10											x					
GalaXMc 71	S5	S25											...					
GalaXMc 72	S2	S25											...					
GalaXMc 74	S5	S25											...					
GalaXMc 75	S2	S10											x					
GalaXMc 76	S2	S25											...					
FujiXHoney 2	S1	S2	x	x	...	...		x	x		...			...		x	x	...
FujiXHoney 3	S2	S9	...	x	x	...		...	x		x			...		...	x	x
FujiXHoney 5	S2	S9	...	x	x	...		...	x		x			...		...	x	x
FujiXHoney 6	S1	S24	x	...	...	x		x	...		...			x		x	...	...
FujiXHoney 7	S2	S9	...	x	x	...		...	x		x			...		...	x	x
FujiXHoney 8	S9	S24	...	...	x	x		...	...		x			x		...	...	x
FujiXHoney 9	S1	S2	x	x	...	...		x	x		...			...		x	x	...
FujiXHoney 10	S1	S24	x	...	...	x		x	...		...			x		x	...	...
FujiXHoney 12	S1	S24	x	...	...	x		x	...		...			x		x	...	...
FujiXHoney 13	S2	S9	...	x	x	...		...	x		x			...		...	x	x
FujiXHoney 14	S2	S9	...	x	x	...		...	x		x			...		...	x	x
FujiXHoney 15	S1	S24	x	...	...	x		x	...		...			x		x	...	...
FujiXHoney 16	S2	S9	...	x	x	...		...	x		x			...		...	x	x
FujiXHoney 18	S2	S9	...	x	x	...		...	x		x			...		...	x	x
FujiXHoney 19	S2	S9	...	x	x	...		...	x		x			...		...	x	x
FujiXHoney 20	S1	S24	x	...	...	x		x	...		...			x		x	...	...
FujiXHoney 23	S1	S2	x	x	...	...		x	x		...			...		x	x	...
FujiXHoney 26	S9	S24	...	...	x	x		...	...		x			x		...	...	x
FujiXHoney 27	S2	S9	...	x	x	...		...	x		x			...		...	x	x
FujiXHoney 31	S2	S9	...	x	x	...		...	x		x			...		...	x	x
FujiXHoney 32	S1	S24	x	...	...	x		x	...		...			x		x	...	...
FujiXHoney 33	S1	S2	x	x	...	...		x	x		...			...		x	x	...
FujiXHoney 35	S1	S24	x	...	...	x		x	...		...			x		x	...	...
FujiXHoney 36	S1	S2	x	x	...	...		x	x		...			...		x	x	...
FujiXHoney 37	S9	S24	...	...	x	x		...	...		x			x		...	...	x
FujiXHoney 40	S2	S9	...	x	x	...		...	x		x			...		...	x	x
FujiXHoney 45	S1	S2	x	x	...	...		x	x		...			...		x	x	...
FujiXHoney 48	S2	S9	...	x	x	...		...	x		x			...		...	x	x
FujiXHoney 49	S1	S2	x	x	...	...		x	x		...			...		x	x	...
FujiXHoney 50	S9	S24	...	...	x	x		...	...		x			x		...	...	x
FujiXHoney 51	S1	S24	x	...	...	x		x	...		...			x		x	...	...
FujiXHoney 52	S1	S24	x	...	...	x		x	...		...			x		x	...	...
FujiXHoney 53	S1	S2	x	x	...	...		x	x		...			...		x	x	...
FujiXHoney 54	S9	S24	...	...	x	x		...	...		x			x		...	...	x

Individual	S- haplotpe		SFBB14			SFBB16						SFBB17		SFBB19	SFBB20
			S9-	S10-	S25-	S1-	S2-	S3-	S10-	S24-	S25-	S10-	S25-	S9-	S28-
			Sau3AI	HindIII	MwoI	EcoRI	EcoRI	MnlI	Sau3AI	ApoI	MseI	Sau3AI	Sau3AI		
			164 170	110 379	119 370	114 195	309	59	132 400	252	105	173 458	270 585		
GDXRD 4	S3	S28						x						...	x
GDXRD 5	S3	S28						x						...	x
GDXRD 6	S3	S28						x						...	x
GDXRD 7	S2	S9						...						x	...
GDXRD 8	S2	S28						...						...	x
GDXRD 9	S2	S9						...						x	...
GDXRD 11	S3	S9						x						x	...
GDXRD 12	S2	S28						...						...	x
GDXRD 13	S3	S28						x						...	x
GDXRD 15	S3	S9						x						x	...
GDXRD 16	S2	S28						...						...	x
GDXRD 18	S2	S28						...						...	x
GDXRD 20	S3	S9						x						x	...
GDXRD 22	S2	S9						...						x	...
GDXRD 24	S2	S9						...						x	...
GDXRD 25	S2	S9						...						x	...
GDXRD 26	S2	S28						...						...	x
GDXRD 28	S3	S9						x						x	...
GDXRD 29	S3	S9						x						x	...
GDXRD 30	S2	S28						...						...	x
GDXRD 31	S3	S9						x						x	...
GDXRD 32	S3	S9						x						x	...
GDXRD 33	S2	S9						...						x	...
GDXRD 34	S3	S28						x						...	x
GDXRD 35	S3	S9						x						x	...
GDXRD 36	S2	S9						...						x	...
GDXRD 37	S3	S9						x						x	...
GalaXMc 5	S5	S10		x	...				x		...	x	...		
GalaXMc 6	S2	S10		x	...				x		...	x	...		
GalaXMc 7	S2	S25		...	x				...		x	...	x		
GalaXMc 8	S2	S25		...	x				...		x	...	x		
GalaXMc 9	S5	S25		...	x				...		x	...	x		
GalaXMc 10	S5	S10		x	...				x		...	x	...		
GalaXMc 11	S2	S10		x	...				x		...	x	...		
GalaXMc 13	S2	S25		...	x				...		x	...	x		
GalaXMc 14	S5	S10		x	...				x		...	x	...		
GalaXMc 16	S2	S25		...	x				...		x	...	x		
GalaXMc 17	S2	S10		x	...				x		...	x	...		
GalaXMc 18	S2	S25		...	x				...		x	...	x		
GalaXMc 21	S5	S10		x	...				x		...	x	...		
GalaXMc 28	S5	S10		x	...				x		...	x	...		
GalaXMc 29	S2	S10		x	...				x		...	x	...		
GalaXMc 30	S2	S10		x	...				x		...	x	...		
GalaXMc 31	S2	S10		x	...				x		...	x	...		
GalaXMc 36	S5	S25		...	x				...		x	...	x		
GalaXMc 38	S2	S25		...	x				...		x	...	x		
GalaXMc 39	S2	S25		...	x				...		x	...	x		
GalaXMc 40	S5	S10		x	...				x		...	x	...		
GalaXMc 41	S2	S25		...	x				...		x	...	x		
GalaXMc 42	S5	S10		x	...				x		...	x	...		
GalaXMc 44	S2	S25		...	x				...		x	...	x		
GalaXMc 45	S2	S25		...	x				...		x	...	x		
GalaXMc 46	S2	S10		x	...				x		...	x	...		
GalaXMc 47	S5	S10		x	...				x		...	x	...		
GalaXMc 48	S5	S10		x	...				x		...	x	...		
GalaXMc 50	S5	S10		x	...				x		...	x	...		
GalaXMc 51	S5	S25		...	x				...		x	...	x		
GalaXMc 52	S2	S10		x	...				x		...	x	...		
GalaXMc 53	S2	S10		x	...				x		...	x	...		
GalaXMc 54	S5	S10		x	...				x		...	x	...		
GalaXMc 55	S2	S25		...	x				...		x	...	x		
GalaXMc 56	S2	S10		x	...				x		...	x	...		
GalaXMc 57	S2	S10		x	...				x		...	x	...		
GalaXMc 58	S5	S25		...	x				...		x	...	x		
GalaXMc 60	S2	S10		x	...				x		...	x	...		
GalaXMc 63	S5	S25		...	x				...		x	...	x		
GalaXMc 65	S5	S10		x	...				x		...	x	...		
GalaXMc 66	S2	S25		...	x				...		x	...	x		
GalaXMc 67	S2	S25		...	x				...		x	...	x		
GalaXMc 69	S2	S10		x	...				x		...	x	...		
GalaXMc 71	S5	S25		...	x				...		x	...	x		
GalaXMc 72	S2	S25		...	x				...		x	...	x		
GalaXMc 74	S5	S25		...	x				...		x	...	x		
GalaXMc 75	S2	S10		x	...				x		...	x	...		
GalaXMc 76	S2	S25		...	x				...		x	...	x		
FujiXHoney 2	S1	S2	...			x	x			...					
FujiXHoney 3	S2	S9	x			...	x			...					
FujiXHoney 5	S2	S9	x			...	x			...					
FujiXHoney 6	S1	S24	...			x	...			x					
FujiXHoney 7	S2	S9	x			...	x			...					
FujiXHoney 8	S9	S24	x			...	...			x					
FujiXHoney 9	S1	S2	...			x	x			...					
FujiXHoney 10	S1	S24	...			x	...			x					
FujiXHoney 12	S1	S24	...			x	...			x					
FujiXHoney 13	S2	S9	x			...	x			...					
FujiXHoney 14	S2	S9	x			...	x			...					
FujiXHoney 15	S1	S24	...			x	...			x					
FujiXHoney 16	S2	S9	x			...	x			...					
FujiXHoney 18	S2	S9	x			...	x			...					
FujiXHoney 19	S2	S9	x			...	x			...					
FujiXHoney 20	S1	S24	...			x	...			x					
FujiXHoney 23	S1	S2	...			x	x			...					
FujiXHoney 26	S9	S24	x			...	...			x					
FujiXHoney 27	S2	S9	x			...	x			...					
FujiXHoney 31	S2	S9	x			...	x			...					
FujiXHoney 32	S1	S24	...			x	...			x					
FujiXHoney 33	S1	S2	...			x	x			...					
FujiXHoney 35	S1	S24	...			x	...			x					
FujiXHoney 36	S1	S2	...			x	x			...					
FujiXHoney 37	S9	S24	x			...	...			x					
FujiXHoney 40	S2	S9	x			...	x			...					
FujiXHoney 45	S1	S2	...			x	x			...					
FujiXHoney 48	S2	S9	x			...	x			...					
FujiXHoney 49	S1	S2	...			x	x			...					
FujiXHoney 50	S9	S24	x			...	...			x					
FujiXHoney 51	S1	S24	...			x	...			x					
FujiXHoney 52	S1	S24	...			x	...			x					
FujiXHoney 53	S1	S2	...			x	x			...					
FujiXHoney 54	S9	S24	x			...	...			x					

* indicate the enzymes used in GalaxRD. # indicate the enzymes used in FujixHoney

Supplementary Table S9. Primers used to characterized the *S-RNase* in the progeny from the crosses 'Golden Delicious' (*S2, S3*) × 'Red Delicious' (*S9, S28*), 'McIntosh' (*S10, S25*) × 'Gala' (*S2, S5*), and 'Fuji' (*S1, S9*) × 'Honeycrisp' (*S2, S24*).

S-RNase	Primer		Amplification	Annealing	Restriction	
	Forward	Reverse	product (bp)	temperature (ºC)	Enzyme	Pattern
'Golden Delicious' × 'Red Delicious'						
S2	CTCTAATCCTACTCCTTG	TATTCTTTTGGCACTTGA	578	49	*Rsa*I	431; 147
S3	AAGTTGTTTACGGTTCAY	ATGTTTACGCCACTGTTT	1476	51		
S9	CTCTAATCCTACTCCTTG	TATTCTTTTGGCACTTGA	575	49	*Rsa*I	312; 116; 107;40
S28	CTCTAATCCTACTCCTTG	TTTGTTTCTGGGTTATGT	471	49	*Rsa*I	173;164;116; 18
'McIntosh' × 'Gala'						
S2	CTCTAATCCTACTCCTTG	TTTGTTTCTGGGTTATGT	449	49		
S5	GTTGTTTACGGTTCACGG	AGTTTTGGTTCCTTATCG	1850	48		
S10	AAGTTGTTTACGGTTCAT	ATGTTTACGCCACTGTTT	1904	51		
S25	TTACGGTTCACGGTTTGT	TGCCCACTGTTTACTCCA	2878	50		
'Fuji' × 'Honeycrisp'						
S1	ATGGTGACGGGGATGATA	GGGAAGACGCACAAGAGC	702	52		
S2	CTCTAATCCTACTCCTTG	TTTGTTTCTGGGTTATGT	449	49	*Rsa*I	431;18
S9	CTCTAATCCTACTCCTTG	TTTGTTTCTGGGTTATGT	446	49	*Rsa*I	312;116;18
S24	RTTGTTTACKGTTCAYGG	TTTCTCCCACTGTTTACG	515	48		

Underlined are the size of the diagnostic bands

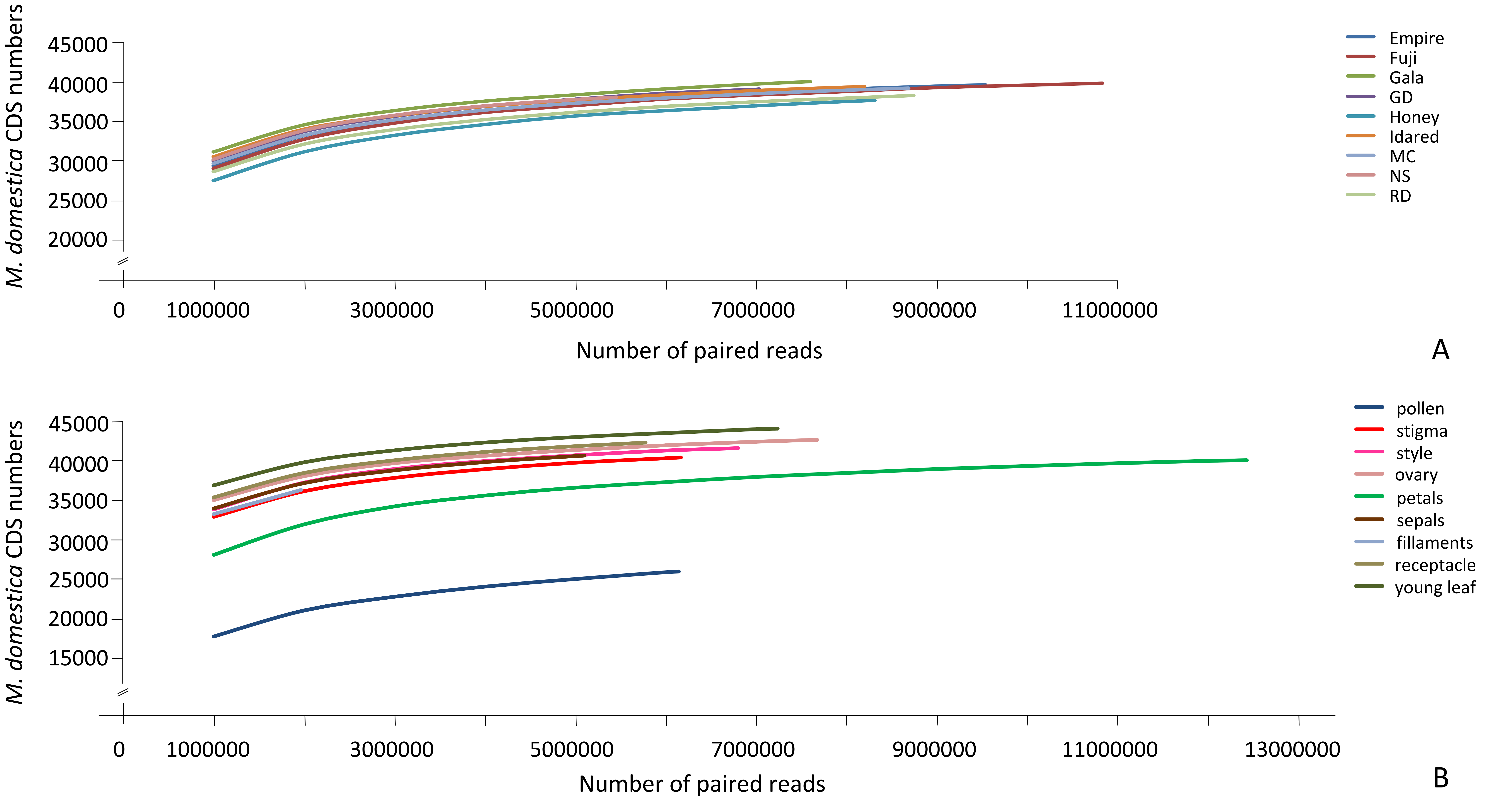

Supplementary Fig. S1

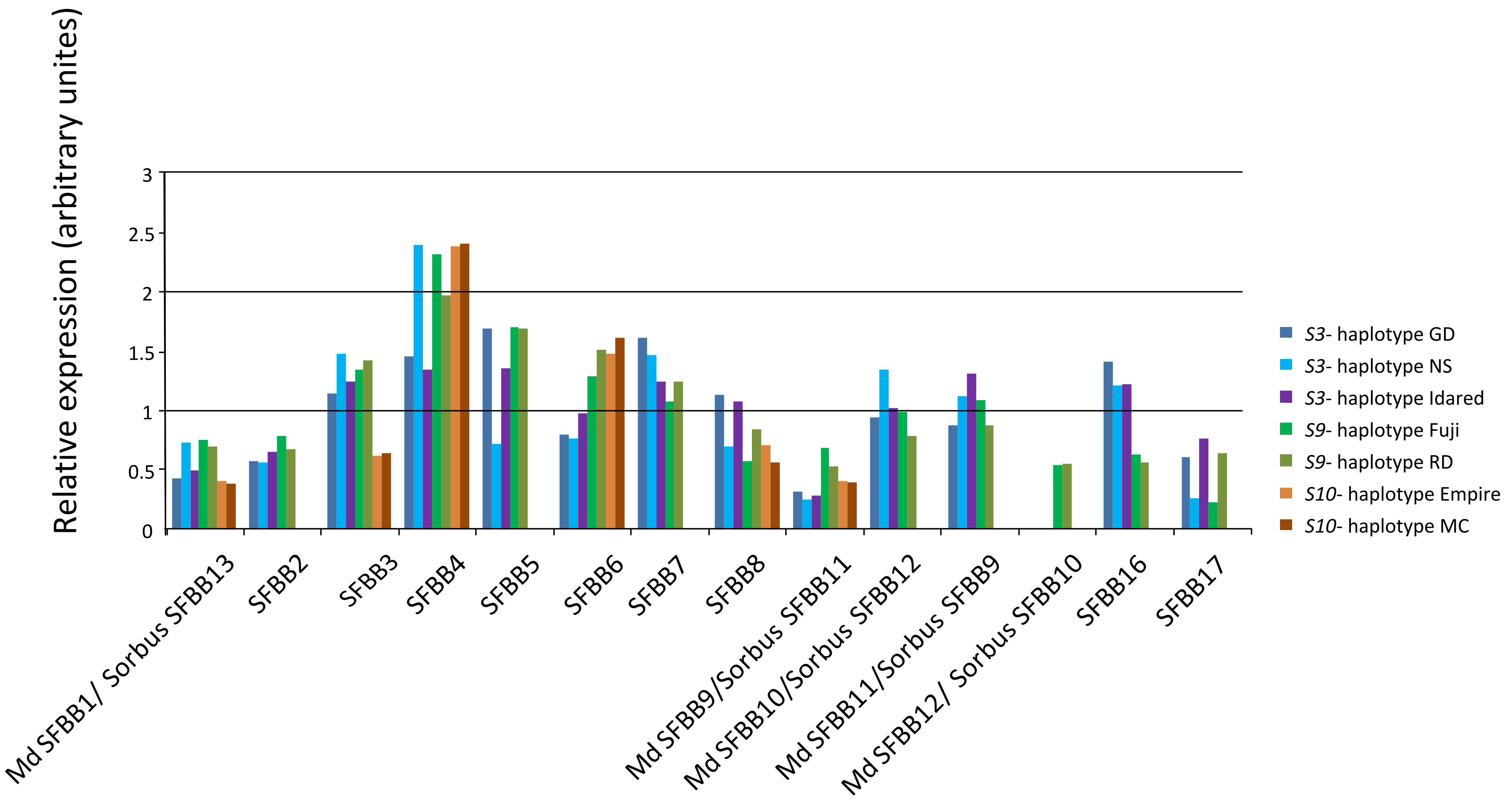

Supplementary Figure S2

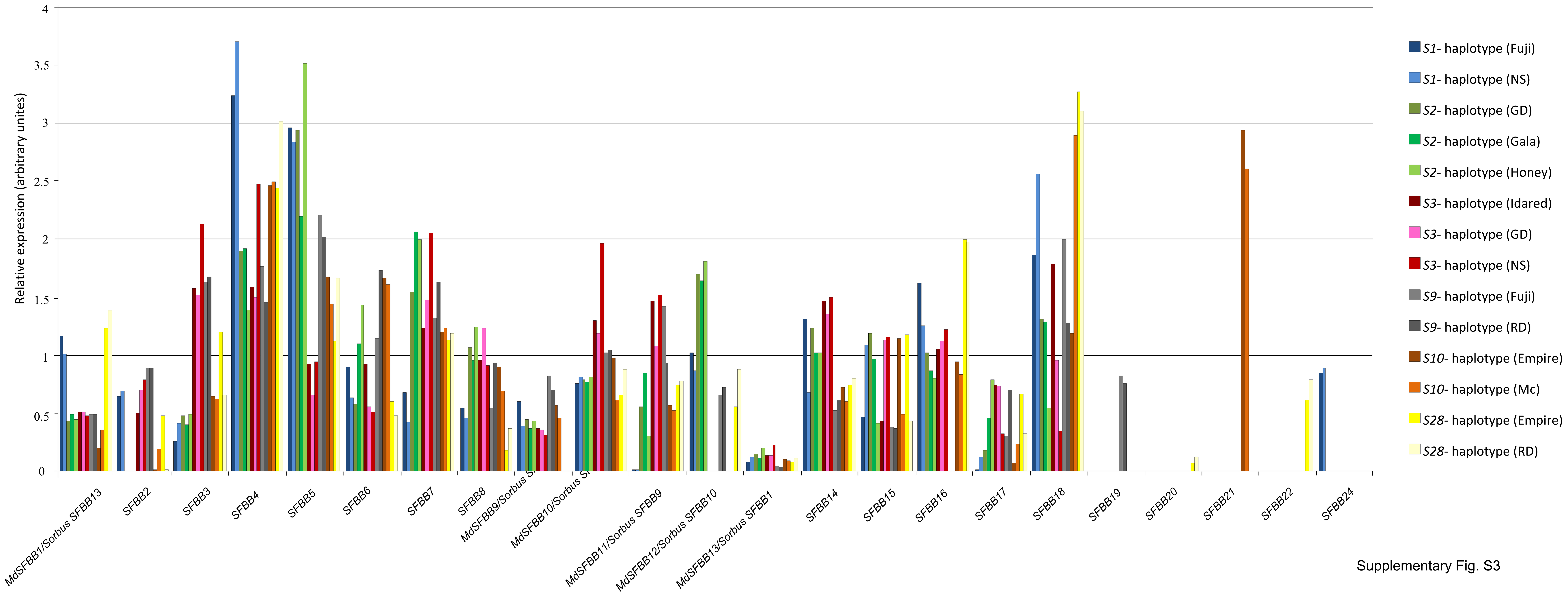

Supplementary Fig. S3

```
                         10        20        30        40        50        60        70        80        90       100
S1-SFBB4       C  MSQVRETETPEDRVVAIMSKLPPKSLMRFKCIRKSWCTLINNPSFVAKHLSNSVDNNFSSYTCILLNRSQVHVFPDKSWKHEVLWSMINFFNDRVSRTLY
S1-SFBB4       M  MSQVRETETPEDRVVAIMSKLPPKSLMRFKCIRKSWCTLINNPSFVAKHLSNSVDNNFSSYTCILLNRSQVHVFPDKSWKHEVLWSMINFFNDRVSRTLY
S2-SFBB4       C  MFEVRESKTPEDMVVEIMSKLPPKSLMRFKCIRKSWCTLINSPSFVAKHLSNSMDNKFSSSTCILLNRSHVHVFPDKSWKHEVLWSMINLFNDRVAHSLY
S2-SFBB4       M  MFEVRESKTPEDMVVEIMSKLPPKSLMRFKCIRKSWCTLINSPSFVAKHLSNSMDNKFSSSTCILLNRSHVHVFPDKSWKHEVLWSMINLFNDRVAHSLY
S3-SFBB4  (16)C  MSQVSETETPEDRVVAIMSKLPPKSLMRFKCIRKSWCTVINSPSFVAKHLSNSVDNKFSSSTCILLNRSQVHVFPDKSWKHEVLWSMINLFNERVARTLY
S3-SFBB4  (16)M  MSQVSETETPEDRVVAIMSKLPPKSLMRFKCIRKSWCTVINSPSFVAKHLSNSVDNKFSSSTCILLNRSQVHVFPDKSWKHEVLWSMINLFNERVARTLY
S3-SFBB4  (14)C  MSQVSETETPEDRVVAIMSKLPPKSLMRFKCIRKSWCTVINSPSFVAKHLSNSVDNKFSSSTCILLNRSQVHVFPDKSWKHEVLWSMINLFNERVARTLY
S3-SFBB4  (14)M  MSQVSETETPEDRVVAIMSKLPPKSLMRFKCIRKSWCTVINSPSFVAKHLSNSVDNKFSSSTCILLNRSQVHVFPDKSWKHEVLWSMINLFNERVARTLY
S5-SFBB4       C  MSQVRETETPEDRVVAIMSKLPPKSLMRFKCIRKSWCTLINNPSFVAKHLSNSVVNNFSSYTCILLNRSQVHVFPDKSWKHEVLWSMINFFNDRVSRTLY
S5-SFBB4       M  MSQVRETETPEDRVVAIMSKLPPKSLMRFKCIRKSWCTLINNPSFVAKHLSNSVVNNFSSYTCILLNRSQVHVFPDKSWKHEVLWSMINFFNDRVSRTLY
S7-SFBB4       C  MSQVRETETPEDRVVAIMSKLPPKSLMRFKCIRKSWCTLINSPSFVAKHLSNSVDNKLSSYICILLSRSQVNVLPDKSWKQEVLWSMINLFNERVAHSLY
S7-SFBB4       M  MSQVRETETPEDRVVAIMSKLPPKSLMRFKCIRKSWCTLINSPSFVAKHLSNSVDNKLSSYICILLSRSQVNVLPDKSWKQEVLWSMINLFNERVAHSLY
S9-SFBB4       C  MSQVRETETPEDRVVAIMSKLPPKSLMRFKCIRKSWCTLINSPSFVAKHLSNSVDNKFSSSTCILLNRSQVHVFPDKSWKHEVLWSKINFFNERLARSLY
S9-SFBB4       M  MSQVRETETPEDRVVAIMSKLPPKSLMRFKCIRKSWCTLINSPSFVAKHLSNSVDNKFSSSTCILLNRSQVHVFPDKSWKHEVLWSKINFFNERLARSLY
S10-SFBB4      C  MSQVRETETPEDRVVAIMSKLPPKSLMRFKCISKSWCTLINSPSFVAKHLSNSVDNKFSSSTCILLNRSQVHVFPDKSWKHEVLWSMINLFNDRLSRSLY
S10-SFBB4      M  MSQVRETETPEDRVVAIMSKLPPKSLMRFKCISKSWCTLINSPSFVAKHLSNSVDNKFSSSTCILLNRSQVHVFPDKSWKHEVLWSMINLFNDRLSRSLY
S24-SFBB4 (17)C  MSQVRETETLEDRVVAIMSKLPPKSLMRFKCIRKSWCTVINSPSFVAKHLSNSVDNKFSSYIFILLNRSQVHVFPDKSWKHEVLWSMINLFNDRVACTLY
S24-SFBB4 (17)M  MSQVRETETLEDRVVAIMSKLPPKSLMRFKCIRKSWCTVINSPSFVAKHLSNSVDNKFSSYIFILLNRSQVHVFPDKSWKHEVLWSMINLFNDRVACTLY
S24-SFBB4 (16)C  MSQVRETETLEDRVVAIMSKLPPKSLMRFKCIRKSWCTVINSPSFVAKHLSNSVDNKFSSYIFILLNRSQVHVFPDKSWKHEVLWSMINLFNDRVACTLY
S24-SFBB4 (16)M  MSQVRETETLEDRVVAIMSKLPPKSLMRFKCIRKSWCTVINSPSFVAKHLSNSVDNKFSSYIFILLNRSQVHVFPDKSWKHEVLWSMINLFNDRVACTLY
S25-SFBB4      C  MSQVSESETPEDKVVEILSKLPPKSLMRFKCIRKSWCTIINSPSFVAKHLSNSVDNKFSSSTCILLNRSQVHVFPDKSWKHEVLWSMIKFFNDRISRTLY
S25-SFBB4      M  MSQVSESETPEDKVVEILSKLPPKSLMRFKCIRKSWCTIINSPSFVAKHLSNSVDNKFSSSTCILLNRSQVHVFPDKSWKHEVLWSMIKFFNDRISRTLY
S28-SFBB4      C  MSQVRETETPEERVVAIMSKLPPKALMRFKCIRRSWCTLINNPSFVAKHLSNSVDNNFSSYTCILLNRSQVHVFPDKSWKHEVLWSMINFFNDRVSRTLY
S28-SFBB4      M  MSQVRETETPEERVVAIMSKLPPKALMRFKCIRRSWCTLINNPSFVAKHLSNSVDNNFSSYTCILLNRSQVHVFPDKSWKHEVLWSMINFFNDRVSRTLY
                  *  * *  * *  ** * ****** *******  **** ** ***********  *  **   *** ** * * ****** ******** ** *    **
                                                                        #     #   #
```

```
                          110       120       130       140       150       160       170       180       190       200
S1-SFBB4        C YNVEDLNIPFPRDDHEHILIHGYCNGIVCVISGKNILLCNPATREFRQLPDSFLLLPSPLGGKFELETDFGGLGFGYDCRAKDYKVVRIIENCEYSDDER
S1-SFBB4        M YNVEDLNIPFPRDDHEHILIHGYCNGIVCVISGKNILLCNPATREFRQLPDSFLLLPSPLGGKFELETDFGGLGFGYDCRAKDYKVVRIIENCEYSDDER
S2-SFBB4        C YNAEDLNIPFPRDDHQHVIIHGYCNGIVCVISGKNILLCNPATREFRQLPDSFLLLPSPLGGKFELETDFGGLGFGYDCKAKDYKVVRIIENCEYSDDER
S2-SFBB4        M YNAEDLNIPFPRDDHQHVIIHGYCNGIVCVISGKNILLCNPATREFRQLPDSFLLLPSPLGGKFELETDFGGLGFGYDCKAKDYKVVRIIENCEYSDDER
S3-SFBB4    (16)C YDVEDLNIPFPRDDHQHVLIHGYCNGIVCVISGKNILLCNPATREFRQLPDSFLLLPSPLGGKFELETDFGGLGFGYDCRAKDYKIVRIIENCEYSDDER
S3-SFBB4    (16)M YDVEDLNIPFPRDDHQHVLIHGYCNGIVCVISGKNILLCNPATREFRQLPDSFLLLPSPLGGKFELETDFGGLGFGYDCRAKDYKIVRIIENCEYSDDER
S3-SFBB4    (14)C YDVEDLNIPFPRDDHQHVLIHGYCNGIVCVISGKNILLCNPATREFRQLPDSFLLLPSPLGGKFELETDFGGLGFGYDCRAKDYKIVRIIENCEYSDDER
S3-SFBB4    (14)M YDVEDLNIPFPRDDHQHVLIHGYCNGIVCVISGKNILLCNPATREFRQLPDSFLLLPSPLGGKFELETDFGGLGFGYDCRAKDYKIVRIIENCEYSDDER
S5-SFBB4        C YNVEDLNIPFPRDDHEHILIHGYCNGIVCVISGKNILLCNPATREFRQLPDSFLLLPSPLGGKFELETDFGGLGFGYDCRAKDYKVVRIIENCEYSDDER
S5-SFBB4        M YNVEDLNIPFPRDDHEHILIHGYCNGIVCVISGKNILLCNPATREFRQLPDSFLLLPSPLGGKFELETDFGGLGFGYDCRAKDYKVVRIIENCEYSDDER
S7-SFBB4        C YDVEDLNIPFPRDDHQHVLIHGYCNGIVCVISGKNILLCNPATREFRQLPDSFLLLPSRLGGKFELETDFGRLGFGYDCRAKDYKVVRIIENCEYSDDER
S7-SFBB4        M YDVEDLNIPFPRDDHQHVLIHGYCNGIVCVISGKNILLCNPATREFRQLPDSFLLLPSRLGGKFELETDFGRLGFGYDCRAKDYKVVRIIENCEYSDDER
S9-SFBB4        C YDVEDLNIPFPRDDHQHVLIHGYCNGIVCVISGKNILLCNPATREFRQLPDSFLLLPSPLGGKFELETDFGGLGFGYDCRAKDYKVVRIIENCEYSDDER
S9-SFBB4        M YDVEDLNIPFPRDDHQHVLIHGYCNGIVCVISGKNILLCNPATREFRQLPDSFLLLPSPLGGKFELETDFGGLGFGYDCRAKDYKVVRIIENCEYSDDER
S10-SFBB4       C YDVEDLNIPFPRDDHQHVLIHGYCNGIVCVISGKNILLCNPATREFRQLPDSFLLLPSPLGGKFELETDFGGLGFGYDCRAKDYKVVRIIENCEYSDDER
S10-SFBB4       M YDVEDLNIPFPRDDHQHVLIHGYCNGIVCVISGKNILLCNPATREFRQLPDSFLLLPSPLGGKFELETDFGGLGFGYDCRAKDYKVVRIIENCEYSDDER
S24-SFBB4   (17)C YDVDDLNIPFPRDDHQHVLIHGYCNGIVCVISGKNILLCNPATREFRQLPDSFLLLPSPLGGKFELETDFGGLGFGYDCRAKDYKVVRIIENCEYSDDER
S24-SFBB4   (17)M YDVDDLNIPFPRDDHQHVLIHGYCNGIVCVISGKNILLCNPATREFRQLPDSFLLLPSPLGGKFELETDFGGLGFGYDCRAKDYKVVRIIENCEYSDDER
S24-SFBB4   (16)C YDVDDLNIPFPRDDHQHVLIHGYCNGIVCVISGKNILLCNPATREFRQLPDSFLLLPSPLGGKFELETDFGGLGFGYDCRAKDYKVVRIIENCEYSDDER
S24-SFBB4   (16)M YDVDDLNIPFPRDDHQHVLIHGYCNGIVCVISGKNILLCNPATREFRQLPDSFLLLPSPLGGKFELETDFGGLGFGYDCRAKDYKVVRIIENCEYSDDER
S25-SFBB4       C YDVEDLNIPFPRDDHQHVLIHGYCNGIVCVISGKNILLCNPATREFRQLPDSFLVLPSPLSGKFELETDLGGLGFGYDCRAKDYKVVRIIENCEYSDDER
S25-SFBB4       M YDVEDLNIPFPRDDHQHVLIHGYCNGIVCVISGKNILLCNPATREFRQLPDSFLVLPSPLSGKFELETDLGGLGFGYDCRAKDYKVVRIIENCEYSDDER
S28-SFBB4       C YNVEDLNIPFPRDDHEHILIYGYCNGIVCVILGKNILLCNPATREFRQLPDSFLLLPSPLGGKFELETDFGGLGFGYDCRAKDYKVVRIIENCEYSDDER
S28-SFBB4       M YNVEDLNIPFPRDDHEHILIYGYCNGIVCVILGKNILLCNPATREFRQLPDSFLLLPSPLGGKFELETDFGGLGFGYDCRAKDYKVVRIIENCEYSDDER
                  *   *********** *  * ********** ********************** *** * ******** * ******* ***** **************
                             #    # #            #                           ##       # #                #
```

```
                          210       220       230       240       250       260       270       280       290       300
S1-SFBB4      C  TYYHRIPLPHTAEVYTMATNSWKEIKIDISSKTYPCSCSVYLKGFCYWFTRDGEEFILSFNLGDERFHRIQLPSRRESGFEFYYIFVCNESIASFCSLYD
S1-SFBB4      M  TYYHRIPLPHTAEVYTMATNSWKEIKIDISSKTYPCSCSVYLKGFCYWFTRDGEEFILSFNLGDERFHRIQLPSRRESGFEFYYIFVCNESIASFCSLYD
S2-SFBB4      C  TYYHRIPLPHTAEVYTMAANSWKEIKIDISNKTYPCSCSVYLKGFCYWFTRDGEEFILSFDLGDERFHRIQLPSRRESGLEFYYIFLCNESIASFCSLYD
S2-SFBB4      M  TYYHRIPLPHTAEVYTMAANSWKEIKIDISNKTYPCSCSVYLKGFCYWFTRDGEEFILSFDLGDERFHRIQLPSRRESGLEFYYIFLCNESIASFCSLYD
S3-SFBB4  (16)C  TYYHRIPMPHTAEVFTMATNYWKEIKIDISSKTYPCSCSVYLKGFCYWFTRDGEEFILSFDLGDERFNRIQLPSRRESGLEFYYIFLCNESIASFCSRYD
S3-SFBB4  (16)M  TYYHRIPMPHTAEVFTMATNYWKEIKIDISSKTYPCSCSVYLKGFCYWFTRDGEEFILSFDLGDERFNRIQLPSRRESGLEFYYIFLCNESIASFCSRYD
S3-SFBB4  (14)C  TYYHRIPMPHTAEVFTMATNYWKEIKIDISSKTYPCSCSVYLKGFCYWFTRDGEEFILSFDLGDERFNRIQLPSRRESGLEFYYIFLCNESIASFCSRYD
S3-SFBB4  (14)M  TYYHRIPMPHTAEVFTMATNYWKEIKIDISSKTYPCSCSVYLKGFCYWFTRDGEEFILSFDLGDERFNRIQLPSRRESGLEFYYIFLCNESIASFCSRYD
S5-SFBB4      C  TYYHRIPLPHTAEVYTMATNSWKEVKIDISSKTYPCSCSVYLKGFCYWFTRDGEEFILSFDLGDERFHRIQLPSRRESSFEFYYIFLCNESIASFCSLYD
S5-SFBB4      M  TYYHRIPLPHTAEVYTMATNSWKEVKIDISSKTYPCSCSVYLKGFCYWFTRDGEEFILSFDLGDERFHRIQLPSRRESSFEFYYIFLCNESIASFCSLYD
S7-SFBB4      C  TYYHRIPLPHTAELYTMATNSWKEIKIDISSKTYPCSCSVYLKGFCYWLTRDGEEFILSFDLGDERFHRIQLPSRSEFGLEFYYIFLCNESIASFCSLYN
S7-SFBB4      M  TYYHRIPLPHTAELYTMATNSWKEIKIDISSKTYPCSCSVYLKGFCYWLTRDGEEFILSFDLGDERFHRIQLPSRSEFGLEFYYIFLCNESIASFCSLYN
S9-SFBB4      C  TYYHRIPLPHTAEVYTMATNSWKEIKIDISSKTYPCSCSVYLKGFCYWFTRDGEEFILSFDLGDERFHRIQLPSRKESGFEFYYIFLCNESIASFCSLYD
S9-SFBB4      M  TYYHRIPLPHTAEVYTMATNSWKEIKIDISSKTYPCSCSVYLKGFCYWFTRDGEEFILSFDLGDERFHRIQLPSRKESGFEFYYIFLCNESIASFCSLYD
S10-SFBB4     C  TYYHRIPLPHTAEVYTMATNSWKEIKIDISSKTYPCSCSVYLKGFCYWFTRDGEEFILSFDLGDERFHRIQLPSRRESGFEFYYIFLCNESIASFCSLYD
S10-SFBB4     M  TYYHRIPLPHTAEVYTMATNSWKEIKIDISSKTYPCSCSVYLKGFCYWFTRDGEEFILSFDLGDERFHRIQLPSRRESGFEFYYIFLCNESIASFCSLYD
S24-SFBB4 (17)C  TYYHRIPLPHTAEVYTMATDSWKEIKIDISSKTYPCSCSVYLKGFCYWFTRDGEEFILSFDLGDERFHRIQLPSRRESGFEFYYIFLCNESIASFCSLYD
S24-SFBB4 (17)M  TYYHRIPLPHTAEVYTMATDSWKEIKIDISSKTYPCSCSVYLKGFCYWFTRDGEEFILSFDLGDERFHRIQLPSRRESGFEFYYIFLCNESIASFCSLYD
S24-SFBB4 (16)C  TYYHRIPLPHTAEVYTMATDSWKEIKIDISSKTYPCSCSVYLKGFCYWFTRDGEEFILSFDLGDERFHRIQLPSRRESGFEFYYIFLCNESIASFCSLYD
S24-SFBB4 (16)M  TYYHRIPLPHTAEVYTMATDSWKEIKIDISSKTYPCSCSVYLKGFCYWFTRDGEEFILSFDLGDERFHRIQLPSRRESGFEFYYIFLCNESIASFCSLYD
S25-SFBB4     C  TYYNRIPLPHTSEVYTMATNSWKEIKIDISRKTYPCSCSVYLKGFCYWFTRDGEEFILSFDLGDERFHRIQLPSRRESGLEFYYIFLCNESIASFCSLYD
S25-SFBB4     M  TYYNRIPLPHTSEVYTMATNSWKEIKIDISRKTYPCSCSVYLKGFCYWFTRDGEEFILSFDLGDERFHRIQLPSRRESGLEFYYIFLCNESIASFCSLYD
S28-SFBB4     C  TYYHRIPLPHTAEVYTMATNSWQEVKIDISSKTYPCSCSVYLKGFCYWFTRDGEEFILSFGLGDERFHRIQLPSRRESSFEFYYIFLCNESIASFCSLYD
S28-SFBB4     M  TYYHRIPLPHTAEVYTMATNSWQEVKIDISSKTYPCSCSVYLKGFCYWFTRDGEEFILSFGLGDERFHRIQLPSRRESSFEFYYIFLCNESIASFCSLYD
                 *** *** *** *  ***   * * ***** ***************** *********** ****** ******* *   ****** ********** *
                                 #              #  #               # #                           #
```

```
                          310       320       330       340       350       360       370       380       390
S1-SFBB4       C  RSQDSKSCEIWVMDD-DGVKSSWTKLLVAGPFKGIEKPLTLWKCDELLMIDTDGRVISYNSGIGYLTYLHIPPIINRVIDSQALIYVESIVPVK
S1-SFBB4       M  RSQDSKSCEIWVMDD-DGVKSSWTKLLVAGPFKGIEKPLTLWKCDELLMIDTDGRVISYNSGIGYLTYLHIPPIINRVIDSQALIYVESIVPVK
S2-SFBB4       C  RSEDSKSCEIWVMDDYDGVKSSWTKLLVAGPFKGIEKPLTLWKCDELLMLATDGRVISYNSSIGYLNYLHIPPIINRIIDSQALIYVESIVSVQ
S2-SFBB4       M  RSEDSKSCEIWVMDDYDGVKSSWTKLLVAGPFKGIEKPLTLWKCDELLMLATDGRVISYNSSIGYLNYLHIPPIINRIIDSQALIYVESIVSVQ
S3-SFBB4  (16)C  RSEDSKSCEIWVMDDYDGVKSSWTKLLVAGPFKGIEKPLTLWKCDELFMIDTDGRVISYNSSIGYLSYLHIPPIINRVIDSQALIYVESIVPIK
S3-SFBB4  (16)M  RSEDSKSCEIWVMDDYDGVKSSWTKLLVAGPFKGIEKPLTLWKCDELFMIDTDGRVISYNSSIGYLSYLHIPPIINRVIDSQALIYVESIVPIK
S3-SFBB4  (14)C  RSEDSKSCEIWVMDDYDGVKSSWTKLLVAGPFKGIEKPLTLWKCDELFMIDTDGRVISYNSSIGYLSYLHIPPIINRVIDSQALIYVESIVPIK
S3-SFBB4  (14)M  RSEDSKSCEIWVMDDYDGVKSSWTKLLVAGPFKGIEKPLTLWKCDELFMIDTDGRVISYNSSIGYLSYLHIPPIINRVIDSQALIYVESIVPIK
S5-SFBB4       C  RSEDSKSCEIWVMDDYDGVKSSWTKLLVAGPFKGIEKPLTLWKCDELLMIDTDGRVISYNSGIGYLTYLHIPPIINRVIDSQALIYVESIVPVK
S5-SFBB4       M  RSEDSKSCEIWVMDDYDGVKSSWTKLLVAGPFKGIEKPLTLWKCDELLMIDTDGRVISYNSGIGYLTYLHIPPIINRVIDSQALIYVESIVPVK
S7-SFBB4       C  RSEDSKSCEIWIMDDFDGVKSSWTKLLVAGPFKGIEKPLTLWKCDELLMLATDGRVISYNSNIGYLNYLHIPPIINRVIDSQALIYVESIVPVK
S7-SFBB4       M  RSEDSKSCEIWIMDDFDGVKSSWTKLLVAGPFKGIEKPLTLWKCDELLMLATDGRVISYNSNIGYLNYLHIPPIINRVIDSQALIYVESIVPVK
S9-SFBB4       C  RSEDSKSCEIWVMDDYDGVKSSWTKLLVAGPFKGIEKPLTLWKGDELLMIDTNGRVISYNSGIGYLTYLHIPPIINRVIDSQVLIYVESIVPIK
S9-SFBB4       M  RSEDSKSCEIWVMDDYDGVKSSWTKLLVAGPFKGIEKPLTLWKGDELLMIDTNGRVISYNSGIGYLTYLHIPPIINRVIDSQVLIYVESIVPIK
S10-SFBB4      C  RSEDSKSCEIWVMDD-DGVKSSWTKLLVAGPFKGIEKPLTLWKCDELLMIDTDGRVISYNSGIGYLTYLHIPPIINRVIDSQALIYVESIVPVK
S10-SFBB4      M  RSEDSKSCEIWVMDD-DGVKSSWTKLLVAGPFKGIEKPLTLWKCDELLMIDTDGRVISYNSGIGYLTYLHIPPIINRVIDSQALIYVESIVPVK
S24-SFBB4 (17)C  RSQDSKSCEIWVMDDYDGVKSSWTKLLVAGPFKGIEKPLTLWKCDELLMIDTDGRVISYNSSIGYLSYLHIPPIINRVIDSQALIYVESIVPVK
S24-SFBB4 (17)M  RSQDSKSCEIWVMDDYDGVKSSWTKLLVAGPFKGIEKPLTLWKCDELLMIDTDGRVISYNSSIGYLSYLHIPPIINRVIDSQALIYVESIVPVK
S24-SFBB4 (16)C  RSQDSKSCEIWVMDDYDGVKSSWTKLLVAGPFKGIEKPLTLWKCDELLMIDTDGRVISYNSSIGYLSYLHIPPIINRVIDSQALIYVESIVPVK
S24-SFBB4 (16)M  RSQDSKSCEIWVMDDYDGVKSSWTKLLVAGPFKGIEKPLTLWKCDELLMIDTDGRVISYNSSIGYLSYLHIPPIINRVIDSQALIYVESIVPVK
S25-SFBB4      C  RSEDSKLCEIWVMDDYDGVKSSWTKLLVAGPFKGIEKPLTLWKCDELLMIDTNGRVISYNSSIGYLSYLHIPLIINRVIDSQALIY--------
S25-SFBB4      M  RSEDSKLCEIWVMDDYDGVKSSWTKLLVAGPFKGIEKPLTLWKCDELLMIDTNGRVISYNSSIGYLSYLHIPLIINRVIDSQALIY--------
S28-SFBB4      C  RSEDSKSCEIWVMDDYDGVKSSWTKLLVAGPFKGIEKPLTLWKCDELLMIDTDGRVISYNSGIGYLTYLHIPPIINRVIDSQALIYVESIVPVK
S28-SFBB4      M  RSEDSKSCEIWVMDDYDGVKSSWTKLLVAGPFKGIEKPLTLWKCDELLMIDTDGRVISYNSGIGYLTYLHIPPIINRVIDSQALIYVESIVPVK
                  ** *** **** *** *************************** *** *  * ******** **** ***** **** **** ***
                    ##
```

Supplementary Fig. S4

```
                                                 10        20        30        40        50        60        70        80        90
S1-RNase (D50837)                       -MVTGMIYVVMMVFSLILLLILSSSTVGFDYYQFTQQYQPAVCNSNPTPCKDPPDKLFTVHGLWPSNSNGNDPEYCKAPPYHTIKM---LE
S2-RNase (U12199)                       MGTTRMVYIVTMLFSLIVLILSSSTVGYDYFQFTQQYQPAACNSNPTPCKDPPDKLFTVHGLWPSNMNRSELFNCSSSNVTYAKIQN-IR
S3-RNase (U12200; AB428425)             MGITGMIYMVTMVFSLIVLILSSSAVKFDYFQFTQQYQPAVCSSNPTPCKDPPDKLFTVHGLWPSNVNGSDPKKCKTTILNPQTITN-LT
S5-RNase (U19791; AB428427)             -------------------------------QFTQQYQPAACKFHHTPCKDPPDKLFTVHGLWPSNFNGPDPENCKVKPTASQTIDTSLK
S7-RNase (U19792; EU427457; AB032246)   MGITGMIYMVTIVFSLIVLLLSSSAARYDYFQFTQQYQLAACNSKPIPCKDPPDKLFTVHGLWPSDSNGHDPVNCSKSTVDAQKLGN-LT
S9-RNase (AB270792; D50836)             MGITGMIYMVTMVFSLIVLILSSPTVGYDYFQFTQQYQPAVCHFNPTPCRDPPDKLFTVHGLWPSNSSGNDPIYCKNTTMNSTKIAN-LT
S10-RNase (AB428428)                    -------------------------------QFTQQYQPAVCNSNPTPCKDPPDKLFTVHGLWPSNVNGSDPKKCKATILNPQTITN-LK
S24-RNase (AF016920, AB050635)          -MGTGMIYMVMMVFSLILLLILPSSTVGFDYYQFTQQYQPAVCNSNPTPCKDPTDKLFTVHGLWPSNSNGNDPKYCNAQQYQTMKI---LE
S25-RNase (AB428431; AB062100)          MGIMGMIYMVMMVFSLIVLILSSSTVGFDYFQFTHQYQPAVCNSNRTPCKDPPDKLFTVHGLWPSNRNGPDPEYCKNTTLDVTKIGN-LQ
S28-RNase (AF201748; AB035273)          MGITGMIYMFTMVFSLIVLILSSSTVGYDYFQFTQQYQPAVCNSNPTPCNDPPEKLFTVHGLWPSNKNGPDPEKCKNIQMNSQKIGN-MA
                                                                       *** *** * *     ** **  ***********          *
                                                                    __________               ____________
                                                                        C1                        C2

                     100       120       130       140       150       160       170       180       190       200       210
S1-RNase    PQLVIIWPNVLNRNDHEGFWRKQWDKHGSCASSPIQNQKHYFDTVIKMYTTQKNVSEILSKANIKPGRKNRPLVDIENAIRNVINNMTPKFKCQKNTRTSLTELVEVGL
S2-RNase    TQLEMIWPNVFNRKNHLGFWNREWNKHGACGYPTIRNDLHYFQTVIKMYITQKNVSDILSKAKIEPDGNIRTQKEIVDAIRKGIHGKEPNLKCQKNTQMT--ELVEVTL
S3-RNase    AQLEIIWPNVLNRKAHARFWRKQWRKHGTCGYPTIADDMHYFSTVIEMYITKKQNVSEILSKAKIKPEKKFRTRDDIVNAISQSIDYKKPKLKCKNNNQIT--ELVEVGL
S5-RNase    PQLEIIWPNVFNRADHESFWQKQWDKHGTCGSPTIIDKNHYFETVIRMYITEKQNVSYILSKANINPDGKGRTRKDIQIAIRNSTNDKEPKLKCQTKNGIT--ELVEVSL
S7-RNase    TQLEIIWPNVYNRTDHISFWDKQWNKHGTCGHPTIMNDIHYFQTVIKMYITQKQNVSKILSRAKIEPEGKPRKQVDIVNAIRKGTNDKEPKLKCQKNNQVT--ELVEVTL
S9-RNase    ARLEIIWPNVLDRTDHITFWNKQWNKHGSCGHPAIQNDMHYLQTVIKMYITQKQNVSEILSKAKIEPVGKFRTQKEIEKAIRKGTNNKEPKLKCQKNSQRT--ELVEVTI
S10-RNase   AQLEIIWPNVLNRRAHVRFWRKQWRKHGACGYPTIADDMHYFSTVIEMYITKKQNVSEILSKAKIKPEGRFRTRDDIVNAISQSIDYKKPKLKCKINNQTT--ELVEVGL
S24-RNase   PQLVIIWPNVLNRNDHEGFWRKQWEKHGSCASSPIQNQKHYFDTVIKMYTTQKNISEILSKANIKPGRKNRTLVDIENAIRNVINNMTPQFKCQKNTRTSLTELVEVGL
S25-RNase   AQLDIIWPNVYDRTNNVGFWSKQWAKHGICGSPTIQDDVNYLETVINLYIIKKQNVFEILSNAKIEPEGKNRTRKDIVKAIRSGTKGKRPKLKCQKNNRTT--ELVEVTL
S28-RNase   AQLEIIWPNVLNRTDHVGFWEREWLKHGTCGYPTIRDDMHYLKTVIKMYITQKQNVSAILAKAMIQPNGQNRSLVDIENAIRSGTNNMKPKFKCQKNTRTT-TELVEVTL
              *  *****  *     **   * *** *    *     *  ***  *   ***   **  * * *         *  **        *  **         *****
                             ______________                      _______________                                ________
                                   C3                                  C4                                          C5

                 220       230       240
S1-RNase    CSDSNLTQFINCPRPFPQGSRNFCPTN-IQY
S2-RNase    CSDGNLKQFIDCPHHFPNGSRHNCPTNHILY
S3-RNase    CSDNNLTQFIDCPRPFPQGSPFFCPTNNIQY
S5-RNase    CSNYFGKNFINCPNKTPGKTRYSCPTNDIHY
S7-RNase    CSNRNLTGFINCPRHIPNGSRYSCPTKNILY
S9-RNase    CSDRNLNQFIDCPRPILNGSRYYCPTNNILY
S10-RNase   -------------------------------
S24-RNase   CSDSNLTQFINCPRPFPRGSRYFCPTN-IQY
S25-RNase   CSDRNLTQLINCPNLIKPKSPYFCPLKSIQY
S28-RNase   CRDRDLTKFINCPQP-PQGSRYLCPAD-VQY

            ___
```

Supplementary Fig. S5
